# Were COVID and the Great Recession well-being reducing?

**DOI:** 10.1371/journal.pone.0305347

**Published:** 2024-11-27

**Authors:** David G. Blanchflower, Alex Bryson

**Affiliations:** 1 Bruce V. Rauner Professor of Economics, Department of Economics, Dartmouth College, Hanover, NH, United States of America; 2 Adam Smith Business School, University of Glasgow, Glasgow, Scotland, United Kingdom; 3 Professor of Quantitative Social Science, Social Research Institute, University College London, London, England, United Kingdom; Indian Institute of Management Shillong, INDIA

## Abstract

Using micro-data on six surveys–the Gallup World Poll 2005–2023, the U.S. Behavioral Risk Factor Surveillance System, 1993–2022, Eurobarometer 1991–2022, the UK Covid Social Survey Panel, 2020–2022, the European Social Survey 2002–2020 and the IPSOS Happiness Survey 2018–2023 –we show individuals’ reports of subjective wellbeing in Europe declined in the Great Recession of 2008/9 and during the Covid pandemic of 2020–2021 on most measures. They also declined in four countries bordering Ukraine after the Russian invasion in 2022. However, the movements are not large and are not apparent everywhere. We also used data from the European Commission’s Business and Consumer Surveys on people’s expectations of life in general, their financial situation and the economic and employment situation in the country. All of these dropped markedly in the Great Recession and during Covid, but bounced back quickly, as did firms’ expectations of the economy and the labor market. Neither the annual data from the United Nation’s Human Development Index (HDI) nor data used in the World Happiness Report from the Gallup World Poll shifted much in response to negative shocks. The HDI has been rising in the last decade reflecting overall improvements in economic and social wellbeing, captured in part by real earnings growth, although it fell slightly after 2020 as life expectancy dipped. This secular improvement is mirrored in life satisfaction which has been rising in the last decade. However, so too have negative affect in Europe and despair in the United States.

## Introduction

"*There are serious problems in using well-being measures for tracking the performance of the economy over time*. *They cannot be expected to change much in response to even historically large changes in macroeconomic activity—the predicted (and actual) effects are just too small*.*"*

Angus Deaton Hicks Lecture [1]

Big economic shocks like the Great Recession of 2008 and giant health shocks like the COVID pandemic of 2020 –which was also an economic shock–are inevitably welfare-reducing. They are labelled negative shocks *because* they are meant to be welfare-reducing economically, financially, and for health and wellbeing reasons. That is not to say that they have no upside. Recessions can generate new growth through creative destruction [2] and reduce mortality rates [3]. Nevertheless, a useful property of a welfare metric is that it should move in response to shocks and move in the right direction–negatively in response to a negative shock, and vice versa. One would also expect such a metric to move more for those who are more adversely (positively) affected by the shock–not necessarily in a monotonic fashion but, still, in a way that can be picked up in the data.

In some instances, one might anticipate responses after a lag, depending on the transmission mechanisms at play. In other cases, we might anticipate short-lived effects, even if the shock is strong, as in the case of the impact of terrorist incidents on momentary wellbeing [4, 5] or to disasters linked to natural hazards like Hurricane Katrina [6]. This does not mean to say that such events are not consequential in the long run. On the contrary, they often are, as indicated by the impact of school shootings. For instance, school shootings lead to drops in student enrollment, a decline in average test scores and an increase in student absenteeism and the likelihood of needing to repeat a grade [7].

Two questions arise: do we expect wellbeing metrics to respond to shocks and, if so, which ones and why? We hypothesize that subjective wellbeing (SWB) metrics capturing positive or negative affect in the short-run are likely to shift in response to a negative shock but only in the short-run. In contrast we argue that metrics requiring individuals to evaluate their lives, such as life satisfaction, and those that elicit their expectations of the future state of affairs in general–the economy, government or democracy, for example–are also liable to shift in response to the business cycle and do so quite markedly as people are asked to evaluate what will happen in the future.

There are potential asymmetries in observed wellbeing in response to shocks. Measures of subjective well-being are more than twice as sensitive to negative as compared to positive economic growth [8]. Easterlin [9] notes that there is an asymmetry in the psychological roots of income evaluations when income is rising versus falling, and this causes a corresponding asymmetry in the response of happiness to income change.

We show individuals’ reports of SWB, such as enjoyment, smiling, sadness, anger, worry and pain in the Gallup World Poll (GWP) do not move as one might have expected in response to two recent major negative shocks, the Great Recession of 2008/9 and the Covid pandemic of 2020/21. Life satisfaction does shift downwards somewhat in response to shocks, but the effects are not large. However, in the case of satisfaction with the economy and democracy the effect of the negative shocks persisted for some time. This was especially apparent when we examine data from the Eurobarometer with multiple surveys each year, which picked up shorter-lived responses.

Using data on both consumers and industrial firms from the European Commission’s Business and Consumer Surveys from 1985–2023 we do find evidence, though, that expectations in relation to the economy, democracy and the labor market moved down sharply in both the Great Recession and during the Covid lockdowns and were predictive of subsequent unemployment.

We show that the United Nations Human Development Index (HDI) capturing country-level economic and social development does not respond very much to the Great Recession of 2008/9 and the Covid pandemic of 2020/21. There is temporal movement in the index, but this tends to follow the pattern of a secular drift upwards. This is not particularly surprising since the sub-indices that go to make up such indices capture institutional features of countries such as their education and welfare systems that, by their nature, move–if at all–rather slowly. The secular rise in the HDI in the last decades reflects overall improvements in economic and social wellbeing.

This secular improvement in the HDI is also mirrored in secular improvements in life satisfaction. However, there have also been secular increases in negative affect in Europe and, in the United States, in despair as captured by the number of poor mental health days in the as captured in Behavioral Risk Factor Surveillance System (BRFSS).

The implication of our paper is that the HDI tracks secular change in underlying welfare over an extended period. Most SWB metrics do not shift in predictable ways in response to macro-shocks and, if they do, the shifts are usually only apparent in the short-term and are not large. In contrast, expectations data respond markedly to downturns but bounce back quite quickly. Secular change in wellbeing is also a feature of the data, but it appears contradictory, with rises in both life satisfaction and negative affect, and despair and bad mental health days in the United States.

## Previous literature

In this section we review the literature on temporal variance in subjective wellbeing and expectations since these are the focus of our empirical analysis. We take them in turn.

### Subjective wellbeing

The literature on temporal variance in wellbeing is long-standing. Much of the research on shocks relies on event studies tracking aspects of individuals’ wellbeing before and after an event which is unambiguously positive or negative. Individuals experience substantial drops in their SWB having experienced divorce, bereavement, or disability. But, in many instances, the data indicate mean reversion, sometimes over relatively short periods of time, consistent with individuals reverting to ‘set points’ [10]. One explanation for these findings is that people respond to adversity and learn to adapt [11].

Diener et al. [12] examined changes in life satisfaction scores over time in response to changes in marital status, assault, disability, unemployment, and childbirth. They reported that people tend to react as expected to these conditions with increases or decreases in their life satisfaction

“*although they often slowly adapt back toward their former levels over time. For some conditions such as marriage adaptation was complete, whereas for other conditions such as unemployment and severe disability people did not fully adapt even after many years*” (12: 505–506).

The literature on exposure to unfortunate events, such as terrorist incidents [4, 5] or natural disasters [6] is also characterized by mean reversion. Sports fans experience shifts in their short-term wellbeing when their team wins or loses, especially if the result is unexpected [13].

A body of literature exists that tracks temporal change in wellbeing within and across days, weeks and months. There is a substantial amount of variance in SWB *within* day, as indicated by time-use studies using day reconstruction methods [14] and experience sampling methods which finds this variance is linked to the activities people are performing, where they are and who they are with. There is also variance in SWB across days of the week. This literature also identifies substantial variation in wellbeing across weeks and months [15] and there is a lot of variation linked to seasonality [16]. All of this temporal variance would be missed in the absence of high-frequency data. However, negative affect, particularly depression, is less susceptible to temporal variance across days and weeks.

One concern that economists have expressed in the light of such findings–particularly the short-lived effects of negative shocks on SWB–is that SWB is unlikely to impact individuals’ behavior. This concern is what lies behind the quote from Angus Deaton presented at the start of this article. And yet, we know that SWB and changes in SWB can impact individuals’ behavior. Job dissatisfaction predicts quit rates [17], for example, although job-related depression and job-related anxiety are not good predictors of quits [18]. Higher SWB is also causally linked to improved productivity at work [19, 20]. It is also consequential because it captures underlying wellbeing, as indicated by the body’s ability to recover from injury and illness [21] and is correlated with biometric markers of wellbeing like pulse, heart rate and blood pressure [22].

Life satisfaction scores also correlate significantly with physiological variables that are thought to track positive moods [12]. Life satisfaction judgments also converge with the number of good versus bad life events that people can recall in timed periods and with mood reports over 6 weeks. Life satisfaction reported in the final year of college correlated significantly with genuine smiles shown on students’ Facebook pages during their first year in college [23].

The focus in this paper is on temporal variance in SWB with the business cycle and shocks. Di Tella et al. [24, 25] and Bell et al. [26] examined micro data from the Eurobarometer surveys and found that both the inflation and unemployment rates lowered life satisfaction across European countries. The extent of the loss in satisfaction was approximately five times higher from a one percentage point rise in the unemployment rate than it was for an equivalent rise in the inflation rate. The impact of the unemployment rate comes from the drop in wellbeing of the 1% who are unemployed and the impact of a rise in the unemployment rate on everyone else. Subsequently El-Jahel et al. [27] examined the effect of the inflation and unemployment rates using the GWP and found that the unemployment rate had a six times higher impact than inflation on wellbeing measured with Cantril’s Ladder-of-Life [28]. It was four times higher for smiling; five times higher for enjoyment, nine times for sadness and thirteen times for pain.

Using the General Social Survey for the United States O’Connor [29] argued that the Great Recession of 2008/9 led to a sizeable reduction in life satisfaction, so that it hit a 40-year low. The drop is accounted for by income losses and unemployment. Writing at the time of the Great Recession Deaton [30] showed how closely related life satisfaction is to GDP per capita and indeed argues that:

“*reports of life satisfaction, at least on average, may provide a useful summary of the different components of peoples’ capabilities. Some of the results in this paper support that position, more so than I had originally expected. In particular, the very strong global relationship between per capita GDP and life satisfaction suggests that on average people have a good idea of how income, or the lack of it, affects their lives”,* [30, p.69].

Boyce et al. [31] used the British Household Panel Survey (BHPS) in the UK to look at how life satisfaction of UK residents changed after the financial crisis. They found that on average the life satisfaction change across the sample was limited but that individuals experiencing unemployment, who lost income, and those who were sick or disabled, experienced the greatest wellbeing reductions.

Others suggest that the Great Recession may have led to subtle but long-lasting effects on SWB. Zhang et al [32] examined distress in the UK from 1991–2019 and noted that improvements in life expectancy stalled after the Great Recession. They found evidence that psychological distress, measured as the GHQ-12 score, worsened after 2015 as did Zhou and Khan [33].

The COVID pandemic which began in early 2020 was both a massive health shock and an economic shock via its direct impact on workers’ health and its effects both on consumer demand and the mobility of workers, consumers and producers. In the UK the UCL Covid Social Study, which ran from 2020–2022, found a big drop in life satisfaction in March 2020, which only slowly recovered [34].

Greyling and Rossouw [35] investigated the impact on happiness of the unprovoked Russian invasion of Ukraine and Covid-19 and found significant decreases in happiness in both instances. They examined ten countries spanning the Northern and Southern hemispheres using a dataset derived from tweets extracted in real-time to capture underlying sentiment by applying Natural Language Processing (machine learning) methods. From these they constructed daily time-series data to measure happiness (Gross National Happiness (GNH)). They found that while the Covid shock and the invasion caused a decrease in GNH adaptation to previous happiness levels occurred within weeks in both cases.

Easterlin and O’Connor [36] examined life satisfaction during COVID using Eurobarometer data for 25 countries. They split the data into three waves. Wave 1 occurred in March 2020-Summer 2020; Wave 2 in Summer 2020-Summer 2021; and Wave 3 in Summer 2021-Autumn 2022. They argued that *“in every one of the 25 Eurobarometer countries an upsurge in the pandemic has a negative association with life satisfaction in at least one and usually both of the second and third waves*.*”* The authors did not examine the micro data that we analyze below. Instead, they based their analysis on a series of survey reports from the EU Commission. We come to the same conclusion using the micro-data.

In an earlier study for the United States using the monthly data from Household Pulse Surveys which started in April 2020 we found an increase in poor mental health, measured as anxiety, depression, and worry, which tracked the rise in Covid cases [37]. Poor mental health peaked before the spike in COVID cases at the start of 2022. This appears to be related to the fact that the death rate for Covid was declining with the availability of vaccines. In a follow-up with the same data series we examined the rise of long covid, which we estimated 14% of adults had experienced, including 6% who had it at the time of interview [38]. Like Myalgic encephalomyelitis/chronic fatigue syndrome (ME/CFS) long COVID is often characterized by profound tiredness. However, long COVID goes away in some cases unlike ME/CFS [39].

Studies for the United States indicate that rising mental ill-health during COVID was a continuation of a longer trend. Using data from the BRFSS, Villas-Boas et al. [40] analyzed data for 2011–2021 and found a rise in depression risk before and during the COVID-19 pandemic. Daly [41] also documents a rise in psychological distress in the United States. Gagné, Schoon and Sacker [42] note in a study that also used the BRFSS that mental distress doubled in men and women aged 18–34 between 1993 and 2019. Daly and Macchia [43] examined GWP data in 113 countries and found that the prevalence of feelings of emotional distress increased from 25 to 31% between 2009 and 2021. Macchia [44] found using the same data source that physical pain increased all around the world between 2009 and 2021. Lamba and Moffitt [45] show the rise in pain in America occurred principally in the years 2007–2010, the time of the Great Recession.

### Expectations

It has long been recognized that consumers and employers are able to assess the state of product and labor markets in such a way that those expectations are capable of predicting future economic trends [46]. Blanchflower and Bryson [46] found consumer expectations indices from the Conference Board and University of Michigan predicted all six of the last six recessions called by the NBER Business Cycle Dating Committee 6–18 months before the date of the recession. In a similar vein they showed that a 10-point shift in expectations compared to the previous 12 month low predicted the onset of the Great Recession in both the United States and Europe [47]. Similarly, individuals’ fears of national unemployment were good predictors of unemployment 12 months later in 29 European countries over the period 1985–2022 in the presence of country fixed effects and lagged unemployment. Industrial firms’ expectations were similarly predictive [48].

The results are consistent with two, not necessarily mutually exclusive propositions. The first is that economic actors acquire knowledge about the state of the economy from their economic and social interactions with others, some of which experts do not possess. We call this ‘*the economics of walking about*’. The second is that these expectations inform the way these economic actors behave subsequently, such that macro-outcomes shift accordingly. The implication of the economics of walking about is that those expectations begin to shift when economic conditions begin to deteriorate since it is this change in underlying conditions that results in changing expectations.

It seems sensible, therefore, to establish the sensitivity of expectations to economic shocks, and compare this to that of wellbeing data. The literature suggests that expectations can affect wellbeing. For instance, expectations of a better future may make it easier to manage during difficult times. That said, the literature indicates that wellbeing and expectations are only moderately correlated [49].

## Data and estimation

We examine movements in wellbeing in European countries using micro-data on individuals. We also consider movements in the expectations of consumer and industrial firms regarding the economy, the labor market and financial conditions, as captured in the European Commission’s data by month by year by country for the period 1985–2023. In addition, we consider movements in the United Nations Human Development Index (HDI) (https://hdr.undp.org/data-center/human-development-index#/indicies/HDI). We focus especially on movements in these wellbeing data during two recent major negative shocks, the Great Recession of 2008/9 and the Covid pandemic of 2020/21.

We analyze eight sets of data including six micro-surveys at the level of the individual.

1) The Gallup World Polls of 2005–2023 (GWP) where we examine Cantril’s contemporaneous life satisfaction measure and the same measure five years ahead. Here sample sizes are 2.1 million across 167 countries. These data are used in the World Happiness Report, and we also make use of their data of chapter 2 from the 2023 World Happiness Report. We present results for a global sample of countries and then later for a subset of European countries.

2) IPSOS Happiness Surveys 2018–2023 (IPSOS) on happiness across 35 countries with a sample size of just over 100,000 observations.

3) The US Behavioral Risk Factor Surveillance System (BRFSS), 1993–2022 on time series changes in negative affect. We examine respondent’s reports on the number of bad mental health days in the last month. We also focus on changes in the proportion of those who say every day in the past month was a bad mental health day–which accounts for an average of one in twenty of the adult population.

4) The UK Covid Social Survey Panel, March 2020-April 2022 (CSS). A team at University College London conducted a daily panel survey in the UK regarding various aspects of Covid, including life satisfaction and depression (https://www.covidsocialstudy.org/). The main findings of the survey are summarized in a final report [50]

5) Sweeps 1–10 of the biannual European Social Survey from 2002–2020 (ESS). Sample sizes are approximately 450,000.

6) The Eurobarometer Survey series, 1991–2022 (EB). We examine a 4-step life satisfaction measure as well as personal and macroeconomic expectations and how they move over time and are predictive of macroeconomic changes. In total we have 1.8 million respondents.

Plus, the two further aggregate surveys.

7) The European Commission’s monthly Business and Consumer Surveys for EU countries and candidate countries, from January 1985 to April 2023 (EC). We examine expectations a year ahead on employment, unemployment and the economic situation of the country from consumers and industrial firms. We also examine two backward looking measures. Sample sizes are approximately 10,700 month by year by country cells. This is an unbalanced panel. As countries join the EU, they join the survey sometimes a year or so before they actually accede to membership. In 2022 the UK left the survey due to Brexit.

8) Annual data from the Human Development Index for 1990–2021 (HDI) with a sample size of just over 5,500 country-year cells.

We track wellbeing and expectations movements in regression analyses where we condition on country and year fixed effects so as to identify within-country correlations between wellbeing/ expectations and these shocks, as well as examining trends in the longer run. We also condition on the age of respondents, and, in some models, we condition directly on country unemployment rates to net out the effects of the business cycle, thus allowing us to establish year-on-year change having netted out labor market effects. With the CSS Panel we run OLS and panel estimates where the latter incorporates person fixed effects.

We start off looking at surveys of multiple countries and then to the United States and the UK and then finally to Europe. Evidence across all these surveys, as we will show, is broadly consistent.

## Results

### Human Development Index and the World Happiness Report

The HDI is a metric compiled by the United Nations Development Program and used to quantify a country’s "*average achievement in three basic dimensions of human development*: *a long and healthy life*, *knowledge*, *and a decent standard of living*." First launched in 1990 it has been released annually ever since, with the exceptions of 2012 and 2020/21 [51, 52].

The health dimension is assessed by life expectancy at birth, the education dimension is measured with two variables i) mean of years of schooling for adults aged 25 years and more and ii) expected years of schooling for children of school entering age. The standard of living dimension is measured by gross national income per capita. The HDI uses the logarithm of income, to reflect the diminishing importance of income with increasing gross national income. The scores for the three HDI dimension indices are then aggregated into a composite index using geometric means.

**Fig 1:**
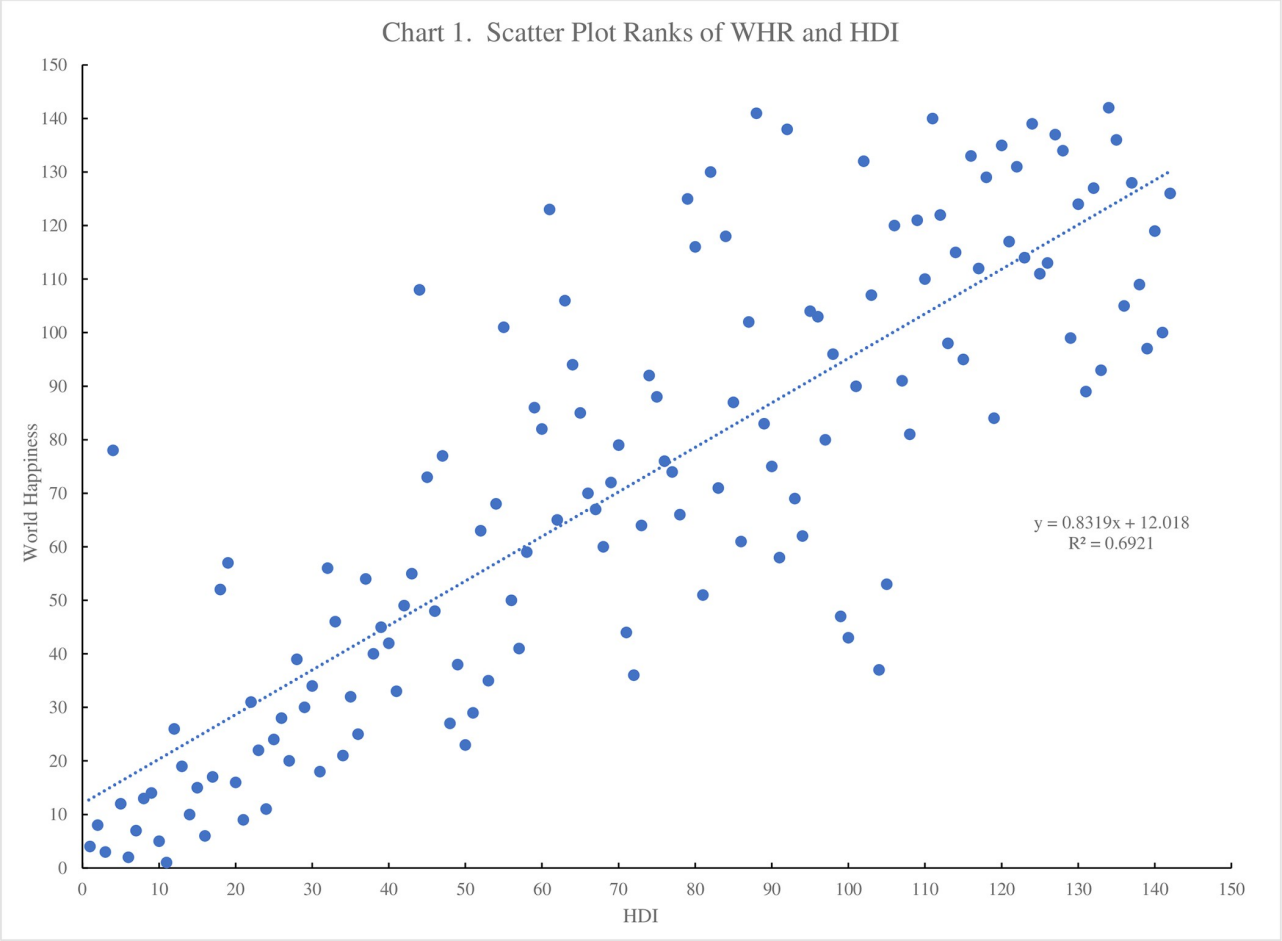
Scatterplot ranks of WHR and HDI.

Fig 1 plots the HDI ranking for 2021 against the 2022 ranking from the World Happiness Report (WHR) for the 142 countries that are ranked in both. The two are highly correlated with an R squared of .69 between the two series. Of the top twenty ranked countries 16 are in both (Switzerland (1,4), Norway (2,8), Iceland (3,3), Denmark (6,2), Sweden (7,7)) with HDI then WHD ranking in parentheses. Fifteen countries are common in the top twenty. Hong Kong is an outlier ranking 4^th^ on HDI and 78^th^ in WHR.

Table 1 shows the result of running regressions on HDI across 191 countries in total for which we have data. These are grouped for eight major regions. We report coefficients on year dummies from regressing the HDI measure on a set for country dummies and a full set of year dummies with 1990 excluded and set to zero. There is some evidence that the HDI did drop during Covid-19 in some regions (such as in Latin America and the Caribbean) but not in others (such as in East Asia). It also declined quite a lot in some countries, such as those in Southern Africa and the rankings of some countries did change quite a bit.

**Table 1:** HDI regressions, 1990–2021.

	Western Europe	Eastern Europe	Western Europe	Latin America	Arab States	East Asia	Sub-Saharan Africa	South Asia
2006	.0966 (26.94)	.0488 (7.55)	.0729 (12.63)	.0827 (21.61)	.0926 (10.99)	.1090 (14.50)	.0573 (11.06)	.1104 (14.74)
2007	.1012 (28.19)	.0581 (8.97)	.0769 (13.32)	.0898 (23.61)	.0995 (11.81)	.1158 (15.41)	.0649 (12.53)	.1166 (15.58)
2008	.1053 (29.36)	.0657 (10.14)	.0799 (13.86)	.0955 (25.11)	.1052 (12.48)	.1206 (16.05)	.0719 (13.89)	.1216 (16.25)
2009	.1061 (29.58)	.0681 (10.52)	.0808 (14.00)	.0977 (25.68)	.1096 (13.01)	.1246 (16.57)	.0795 (15.35)	.1266 (16.91)
2017	.1344 (37.46)	.1111 (17.16)	.1067 (18.49)	.1253 (32.95)	.1323 (15.70)	.1596 (21.40)	.1285 (24.91)	.1872 (25.62)
2018	.1373 (38.27)	.1151 (17.77)	.1096 (19.23)	.1285 (33.78)	.1372 (16.27)	.1632 (21.88)	.1326 (25.71)	.1918 (26.25)
2019	.1413 (39.39)	.1187 (18.33)	.1124 (19.72)	.1303 (34.25)	.1410 (16.72)	.1669 (22.38)	.1370 (26.55)	.1964 (26.88)
2020	.1358 (37.86)	.1084 (16.73)	.1058 (18.57)	.1206 (31.70)	.1326 (15.74)	.1650 (22.12)	.1332 (25.82)	.1925 (26.34)
2021	.1373 (38.27)	.1116 (17.22)	.1087 (19.08)	.1176 (30.91)	.1324 (15.70)	.1619 (21.70)	.1303 (25.26)	.1926 (26.36)
cons	0.7611	0.6601	0.8146	0.6202	0.5854	0.5347	0.8174	0.46
Adj R^2^	0.956	0.9495	0.9306	0.9708	0.9662	0.9606	0.9619	0.9869
N	918	466	400	973	564	676	1311	263

Year dummies from 1991–2005 and 2010–2016 also included, results not reported. The reference year is 1990. All equations include country dummies. T-statistics in parentheses

Region definitions

1. *Arab States* = Algeria; Bahrain; Djibouti; Egypt; Iraq; Jordan; Kuwait; Lebanon; Libya; Morocco; Oman; Palestine; Qatar; Saudi Arabia; Somalia; Sudan; Syria; Tunisia; UAE; Yemen.

2. *East Asia and the Pacific* = Brunei Darussalam; Cambodia; China; Fiji; Indonesia; Kiribati; Korea; Laos; Malaysia; Marshall Islands; Micronesia; Mongolia; Myanmar; Nauru; Palau; Papua New Guinea; Philippines; Samoa; Singapore; Solomon Islands; Thailand; Timor-Leste; Tonga; Tuvalu; Vanuatu; Viet Nam.

3. *Eastern Europe* = Albania; Armenia; Azerbaijan; Belarus; Bosnia and Herzegovina; Georgia; Kazakhstan; Kyrgyzstan; Moldova; Montenegro; North Macedonia; Serbia; Tajikistan; Turkey; Turkmenistan; Ukraine; Uzbekistan.

4. *Western Europe* = Austria; Belgium; Bulgaria; Croatia; Cyprus; Czechia; Denmark; Estonia; Finland; France; Germany; Greece; Hungary; Iceland; Ireland; Italy; Latvia; Lithuania; Luxembourg; Malta; Netherlands; Poland; Portugal; Romania; Slovakia; Slovenia; Spain; Sweden; UK.

5. *Latin America and the Caribbean* = Antigua and Barbuda; Argentina; Bahamas; Barbados; Belize; Bolivia; Brazil; Chile; Colombia; Costa Rica; Cuba; Dominica; Dominican Republic; Ecuador; El Salvador; Grenada; Guatemala; Guyana; Haiti; Honduras; Jamaica; Mexico; Nicaragua; Panama; Paraguay; Peru; Saint Kitts and Nevis; Saint Lucia; Saint Vincent and the Grenadines; Suriname; Trinidad and Tobago; Uruguay; Venezuela.

6. *South Asia and Pacific* = Afghanistan; Bangladesh; Bhutan; India; Iran; Maldives; Nepal; Pakistan; Sri Lanka.

7. *Sub-Saharan Africa* = Angola; Benin; Botswana; Burkina Faso; Burundi; Cabo Verde; Cameroon; CAR; Chad; Comoros; Congo; Congo; Côte d’Ivoire; Equatorial Guinea; Eritrea; Eswatini; Ethiopia; Gabon; Gambia; Ghana; Guinea; Guinea-Bissau; Kenya; Lesotho; Liberia; Madagascar; Malawi; Mali; Mauritania; Mauritius; Mozambique; Namibia; Niger; Nigeria; Rwanda; Sao Tome and Principe; Senegal; Seychelles; Sierra Leone; South Africa; South Sudan; Tanzania; Togo; Uganda; Zambia; Zimbabwe.

8. *Other Western* = Andorra; Australia; Canada; Hong Kong; Israel; Japan; Korea; Liechtenstein; Monaco; New Zealand; Norway; Russian Federation; San Marino; Switzerland; United States.

The HDI moves but slowly. There is no evidence of declines in the size of any of the year dummies in any of the eight regions between 2007 and 2008 or indeed 2009 as recession hit–at the end of 2007 in the US and around April 2008 in most other countries. This is despite the fact that the Great Recession was a major downturn and in many countries the unemployment rate jumped sharply.

If we turn to the COVID shock, there is some evidence that the HDI did drop during Covid-19 in some regions (such as in Latin America and the Caribbean) but not in others (such as in East Asia). More than 90% of the 191 countries analyzed for the 2021/22 HDI report suffered a small decline in the overall HDI in either 2020 or 2021. These declines were largely attributed to the COVID-19 pandemic and its lingering effects. Of note though is these declines had very little effect on country rankings. For example, for the period 2019–2021 in each year Switzerland, Norway and Iceland shared the top three spots. In each of the three years South Sudan was 191^st^, Chad was 190^th^ and Niger was 189^th^.

Table 2 uses data downloaded from the World Happiness Report for all countries—not just Europe—for three measures of wellbeing–Cantril’s ladder of life (Q1) a positive affect variable (Q2) variable and a negative affect variable (Q3) all obtained from the Gallup World Poll [53]. The questions are reproduced below.

*Q1*. *Please imagine a ladder*, *with steps numbered from 0 at the bottom to 10 at the top*. *The top of the ladder represents the best possible life for you and the bottom of the ladder represents the worst possible life for you*. *On which step of the ladder would you say you personally feel you stand at this time*?
*Q2*. *Positive affect is defined as the average of*, *previous day measures for laughter*, *enjoyment*, *and interest all of which are answered yes/not*.
*Q3*. *Negative affect is defined as the average of*, *previous day measures for worry*, *sadness*, *and anger all of which are answered yes/no*.

**Table 2:** Cantril positive and negative affect by country-year cells, 2005–2022.

	Cantril	Positive affect	Negative affect
2005	.2991 (3.21)	.0214 (2.21)	.0114 (1.03)
2006	-.1797 (2.94)	.0003 (0.06)	.0177 (2.46)
2007	-.0121 (0.21)	.0079 (1.29)	.0041 (0.59)
2009	.0556 (0.97)	.0042 (0.72)	.0014 (0.21)
2010	.0227 (0.41)	.0075 (1.29)	-.0055 (0.83)
2011	.0602 (1.11)	.0002 (0.04)	.0005 (0.08)
2012	.0018 (0.03)	.0013 (0.24)	.0136 (2.13)
2013	-.0308 (0.56)	.0140 (2.48)	.0224 (3.47)
2014	-.0045 (0.08)	.0168 (3.00)	.0227 (3.55)
2015	-.0150 (0.28)	.0185 (3.31)	.0284 (4.44)
2016	-.0109 (0.20)	.0179 (3.20)	.0371 (5.79)
2017	.0708 (1.31)	.0076 (1.38)	.0423 (6.66)
2018	.1285 (2.36)	.0130 (2.33)	.0462 (7.20)
**2019**	**.1605 (2.96)**	**.0098 (1.76)**	**.0432 (6.75)**
**2020**	**.1423 (2.49)**	**.0159 (2.70)**	**.0611 (9.08)**
**2021**	**.1031 (1.83)**	**.0068 (1.18)**	**.0466 (7.05)**
2022	.0731 (1.28)	.0053 (0.91)	.0486 (7.21)
Country FE	165	163	164
cons	5.4385	0.6429	0.2457
Adjusted R^2^	0.858	0.8306	0.6698
N	2199	2175	2183

Excluded category 2008. All equations include full set of country dummies. T-statistics in parentheses.

Source: data from Table 2.1 of the World Happiness Report, 2023.

https://worldhappiness.report/ed/2023/#appendices-and-data

Table 2 shows the year dummies from equations that also include country fixed effects. This builds on a set of regressions reported in Helliwell et al [53] but their analysis does not include country fixed effects or report year dummies. We see no evidence of any big change in Cantril, positive or negative affect around 2008, the reference year, which is the year the Great Recession hit. Instead, what is notable is the recent increase in wellbeing captured by Cantril since 2017, something that reversed during Covid. There has also been some growth in negative affect since around 2012 which increased further, albeit temporarily, in 2020 with the advent of Covid. Positive affect has been much flatter in the last two decades but surprisingly actually *rose* in 2020 compared to 2019.

Table 3 supplements the regression in Table 2 with the half dozen variables that Helliwell et al [53] used as controls, together with a lagged dependent variable. These additional variables perform as one might have expected. For example, increases in GDP per capita, freedom and social support are associated with higher Cantril and positive affect scores and lower negative affect. Again, changes in these SWB measures around the time of the Great Recession are not statistically significant. The changes around the time of COVID are also small: there is a small decline in Cantril and a small increase in negative affect in 2020.

**Table 3:** WHR, Cantril positive and negative affect with WHR controls by country-year cells.

	Cantril	Positive Affect	Negative Affect
Lagged dependent variable	.4838 (20.77)	.3314 (14.94)	-.0744 (2.79)
2006	-.5346 (2.66)	-.0049 (0.22)	.0363 (1.37)
**2007**	**-.0168 (0.33)**	**.0048 (0.86)**	**.0065 (0.97)**
**2009**	**-.0081 (0.17)**	**.0038 (0.72)**	**.0087 (1.35)**
2010	-.0964 (2.04)	.0040 (0.77)	.0061 **(**0.97**)**
2011	-.0731 (1.58)	-.0052 (1.03)	.0151 (2.44)
2012	-.1106 (2.35)	-.0000 (0.02)	.0235 (3.76)
2013	-.1567 (3.33)	.0104 (1.99)	.0321 (5.12)
2014	-.1448 (3.05)	.0046 (0.87)	.0352 (5.57)
2015	-.1430 (2.98)	.0054 (1.03)	.0367 (5.76)
2016	-.1690 (3.44)	.0024 (0.46)	.0536 (8.22)
2017	-.0573 (1.15)	-.0064 (1.18)	.0587 (8.87)
2018	-.0667 (1.31)	.0008 (0.14)	.0662 (9.77)
2019	-.0742 (1.45)	-.0032 (0.56)	.0636 (9.32)
2020	-.0952 (1.78)	.0028 (0.47)	.0765 (10.76)
2021	-.1147 (2.19)	-.0094 (1.61)	.0527 (7.536
2022	-.1347 (2.48)	-.0099 (1.66)	.0558 (7.72)
Log GDP per capita*	.2710 (5.26)	.0250 (4.52)	-.0181 (2.71)
Social support*	1.8724 (11.01)	.0663 (3.49)	-.3531 (15.42)
Healthy life expectancy*	-.0163 (2.50)	-.0000 (0.06)	.0039 (14.55)
Freedom to make choices*	.6219 (4.88)	.0959 (6.80)	-.0651 (3.84)
Generosity*	.3680 (3.35)	.0991 (8.13)	.0857 (5.85)
Perceptions of corruption*	-.6551 (4.59)	.0141 (0.90)	.1140 (6.03)
Country FE	153	153	153
cons	-0.3496	0.0691	0.453
Adjusted R^2^	0.9172	0.8875	0.7434
N	1852	1835	1839

Variables defined in Helliwell et al [53]. The reference year is 2008. We lose 2005 due to the introduction of lagged dependent variables. T-statistics in parentheses.

Diener and Tay [54] examined data from the GWP 2005–2014 and ranked countries using a Social Wellbeing metric that included the Cantril ladder variable plus enjoyment, anger sadness and stress. The rankings are presented in S1 Table along with rankings of other variables that also do not move much over time, including economic and material quality of life, physical health, a healthy environment, social quality of life and equality. The rankings by physical health are especially weakly correlated with social wellbeing (r = .26). Denmark for example, ranks 1^st^ on Social Wellbeing and 47^th^ on physical health. Singapore ranks top on physical health and 54^th^ on Social Wellbeing.

It appears that, based on these data from HDI and WHR, the Great Recession and the Covid epidemic do not appear to have been wellbeing reducing.

### IPSOS Happiness Surveys, 2018–2023

IPSOS have kindly granted us access to five of their recent individual level surveys on happiness for 2018–2021 and 2023 [55] There are thirty-five countries of which twelve are from Europe—Belgium, France, Germany, Hungary, Italy, Netherlands, Poland, Romania, Serbia, Spain, Sweden, Turkey, UK. In each of the five surveys a happiness question was asked. There was no survey in 2022.

*Q10*. *Taking all things together*, *would you say you are*: *Very happy (= 4)*, *rather happy (= 3)*, *not very Happy (= 2)*, *not happy at all (= 1)*.

The mean of this variable dropped slightly in 2020 and especially so in the 22 non-European countries. It recovered in 2021, as indicated in Table 4 below.

**Table 4:** Mean Happiness, IPSOS, 2018–2023.

	Europe	Non-Europe
2018	2.72	2.85
2019	2.76	2.79
2020	2.71	2.71
2021	2.78	2.8
2023	2.75	2.94
Total	2.75	2.82

In Table 5 we report the resulting year dummies from a 4-step happiness regression overall and separately for European and non-European countries. Controls also include age and its square and gender and country dummies. There is a notable drop in happiness in 2020 and especially so outside Europe. However, happiness recovers quickly such that, by 2023, it is substantially and significantly much more positive than it was in 2018.

**Table 5:** Happiness in the IPSOS Happiness Surveys, 2018–2023.

	All	Europe	Non-Europe
2019	-.0133 (1.79)	.0518 (4.56)	-.0546 (5.60)
2020	-.0888 (11.90)	-.0319 (2.86)	-.1298 (13.01)
2021	.0196 (2.40)	.0864 (6.97)	-.0243 (2.24)
2023	.1335 (11.85)	.0765 (4.27)	.1567 (10.81)
Cons	2.5948	2.9266	2.5958
Adjusted R^2^	0.0666	0.0558	0.0726
N	101,236	40,662	60,574

2018 excluded category. All equations include age and its square, gender and country dummies. T-statistics in parentheses

We also ran a series of country level regressions with a set of year dummies with 2020 excluded. We tested whether the 2019 dummy was significantly higher than 2020 and found this to be the case in Australia, Canada, France, Germany, India, Mexico, South Africa, Spain, UK, USA, Chile and Peru.

### Behavioral Risk Factor Surveillance System Surveys, 1993–2022

Turning to the United States, the best micro-data on wellbeing over time is the bad mental health days in the past month contained in BRFSS. The question is as follows.

Q4. “*Now thinking about your mental health*, *which includes stress*, *depression*, *and problems with emotions*, *for how many days during the past 30 days was your mental health not good*?”

The overall mean of this variable is 3.46: two-thirds (68%) of respondents say they suffer no bad mental health days, while a further 11.4% say they suffer between 1 and 3. Overall, 5.3% (n = 492,620) say all thirty days were bad mental health days–what we term ‘despair’. These variables have been used previously [56, 57].

**Fig 2:**
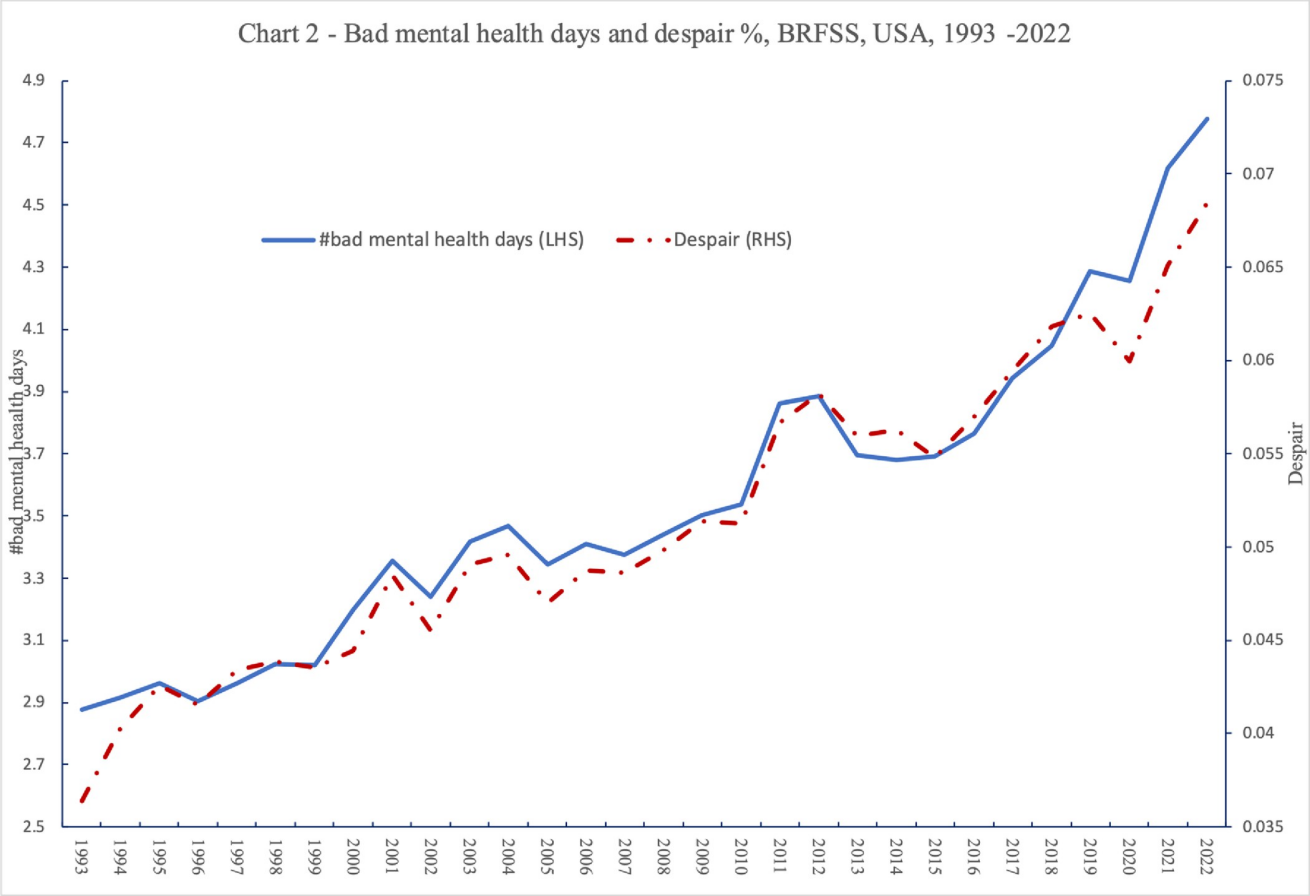
Bad mental health days and % time in despair, BRFSS, USA, 1993–2022.

Table 6 presents within-state trends in bad mental health days relative to 2008 (column 1) together with trends in despair (column 2). Neither metric moves very much during the Great Recession. However, both the number of bad mental health days and despair are rising from 2016, with bad mental health days becoming even more numerous with the Covid-19 outbreak. This is presented graphically in Fig 2.

**Table 6:** #Bad mental health days and despair %, 1993–2023, BRFSS.

	N bad mental health days	% every day is bad mental health day
2005	.0236 (1.32)	-.0034 (0.71)
2006	.0346 (1.94)	.0082 (1.71)
**2007**	-.0380 **(**2.24**)**	-.0061 **(**1.34**)**
**2009**	-.0085 **(**0.50**)**	.0001 **(**0.03**)**
2010	.0770 (4.58)	.0170 (3.75)
2018	.4224 (24.82)	.0565 (12.40)
**2019**	.6110 **(**35.68**)**	.0676 **(**14.81**)**
**2020**	.6914 **(**40.15**)**	.0565 **(**12.26**)**
2021	.8810 (51.65)	.0714 (15.68)
2022	1.0424 (19.97)	.0947 (7.03)
Constant	3.4276	-.1.5593
Adjusted R^2^	0.0085	0.0088
N	9,222,834	9,222,834

All equations include state dummies +Guam, Puerto Rico and US Virgin Islands and gender. Column 1 estimated by OLS. Column 2 estimated by probit. Equations include a full set of year dummies, not all reported. 2008 is the reference year. T-statistics in parentheses

Question. “*Now thinking about your mental health*, *which includes stress*, *depression*, *and problems with emotions*, *for how many days during the past 30 days was your mental health not good*?*”* Despair is where respondent replies “all thirty days”.

### Life satisfaction in the UK Covid Social Survey (CSS) Panel, March 2020-April 2022

From March 2020 a team at University College London conducted a daily panel survey in the UK regarding various aspects of Covid, including life satisfaction and depression (https://www.covidsocialstudy.org/). It has the major benefit that it is a panel of individuals covering responses of around 70,000 individuals. This allows us to control for individual fixed effects. We have daily data that we translate into weeks–as successive seven-day time periods that sometimes overlap months.

One advantage of the survey is that it makes use of the same life satisfaction question used in the UK by the Office of National Statistics in its Annual Population Survey (APS)–a 10-step question.

*Q11*. *Overall*, *how satisfied are you with your life nowadays*?

**Fig 3:**
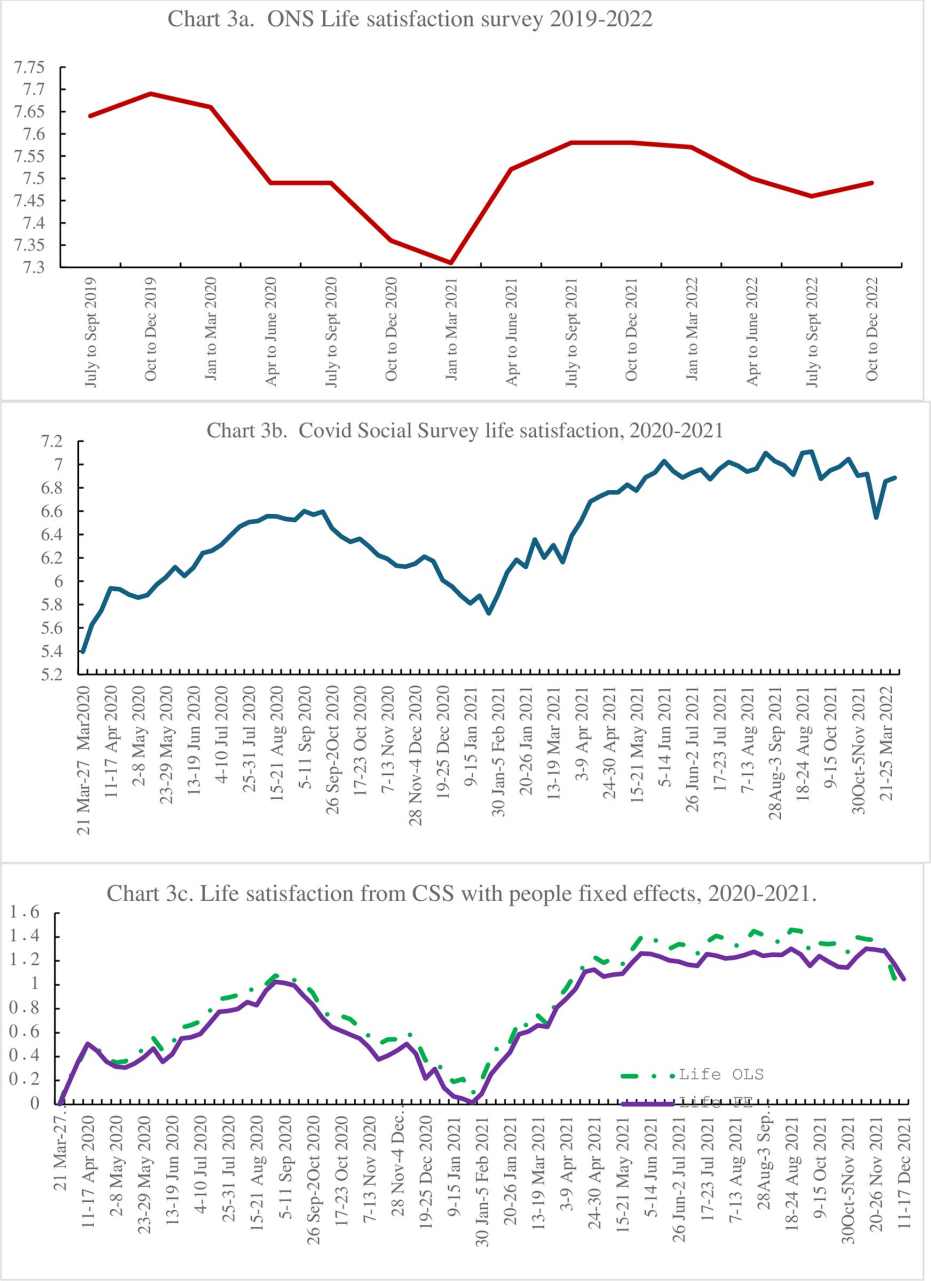
a. ONS Life Satisfaction Survey, 2019–2022, b. Covid Social Survey life satisfaction, 2020–2021, c. Life satisfaction from CSS with person fixed effects, 2020–2021.

This question has the benefit that it has been tracked in the APS over a relatively long time prior to the CSS starting, with both the APS and CSS tracking life satisfaction with the same question since March 2020. The APS life satisfaction data are published quarterly by ONS (https://www.ons.gov.uk/peoplepopulationandcommunity/wellbeing/datasets/quarterlypersonalwellbeingestimatesseasonallyadjusted). The series was started in 2011 and rose steadily from 7.35 in April-June 2011 and was 7.66 in January-March 2020. Fig 3A reports the change in life satisfaction since July-September 2019. It fell with the Covid outbreak to 7.31 in January-March 2021before rising to 7.49 at the end of 2022 seventeen life satisfaction points below its pre-pandemic peak.

The CSS started in the week of 21^st^-27^th^ March 2020 and had a mean of 5.39, much lower than the estimate for the second quarter of the same year from the ONS. Fig 3B plots the time series by week. It peaked at 6.6 in September 2020 and then fell to a low of 5.7 at the start of 2021 before rising through to around 7 with another dip to 6.5 at the start of 2022.

Given the differences in the sample means in the ONS and CSS surveys we reran the CSS estimates incorporating person fixed effects to establish changes in life satisfaction *within person* over time. We regressed the life satisfaction score on the week dummies with controls for age and its square and gender (n = 1,196,311, Adjusted R2 = .0419) and we extracted the week coefficients and plotted them in Fig 3C. We then repeated the exercise dropping the other control variables and including 71,571 people fixed effects (n = 1,196,311, with an adjusted R^2^ of .6920). We extracted those coefficients and plotted them in Fig 3C also. All three figures show a Covid drop in wellbeing and a subsequent pick-up. The results are roughly in line with the Eurobarometer time-series presented below.

We now move on to examine four European data files.

### Gallup World Poll data for Europe, 2005–2023

In Blanchflower and Bryson [58] we estimated US state and country rankings of negative and positive affect using the Gallup World Poll (GWP) and the US Daily Tracker for the period 2010 through 2017. There were no data available from the US Daily Tracker in other years, but the Gallup World Poll has data on several positive affect variables that we examined–Cantril’s 11-step life satisfaction variable question Q1 above, plus three other binary variables–enjoyment, smiling and being well-rested plus four binary negative affect variables–worry, sadness, anger and pain. Smith and Wesselbaum [59] also examined the Cantril ladder variable using the GWP data and found it changed little over time.

In the GWP there are additional data available that we did not examine on what the respondent thinks their life satisfaction is ‘at this time’ and ‘will be in five years’. To be comparable to what has gone before we restrict our analysis to Europe. The mean of life satisfaction in five years (6.8) is slightly higher than Cantril (6.3). This is not simply a sampling issue as this phenomenon is true also in most major advanced countries with the exception of Finland and Japan where they are the same. The reason for this large difference remains unclear.

The overall weighted distributions are presented in Table 7 below. In the case of Cantril’s ladder, 48% scored 6 or less compared with 28% of the life in the future variable.

**Table 7:** Cantril’s ladder and life satisfaction in 5 years, GWP for Europe.

	Cantril	Life in 5
0	1.5	1.6
1	1.4	1.5
2	2.4	2.6
3	4.9	4.4
4	6.6	5.4
5	18.1	11.7
6	13.2	10.2
7	20.1	15.5
8	20.4	23
9	6.8	13.7
10	4.6	10.4
Mean	6.3	6.8
N	590,832	551,655

The starting point for our analysis is the fact that the raw data for Europe on Cantril increased from 2007–2008 and from 2019–2020 as shown below in Table 8.

**Table 8:** Cantril mean score in Europe.

2007	5.39
2008	5.41
2019	5.53
2020	5.64

Table 9 presents a selection of the year dummy coefficients and accompanying t-statistics for nine pooled country regressions for the period 2005–2023, where the reference year is 2008. The models control for country fixed effects, age, age squared and being female. It is striking that Cantril does not perform as one might have expected in response to the two shocks. It plummets between 2006 and 2007 –the years prior to the Great Recession–only to *rise* in 2008 before falling once again in 2009 and 2010. It rises again between 2019 and 2020 at the moment the Covid pandemic erupts, and is followed by an additional rise in 2021, only to fall back in 2022 close to its level in 2019. This is not what one might have expected from a SWB metric responding to two huge negative well-being shocks.

**Table 9:** Gallup 2005–2023 for Europe.

	2006	2007	2009	2010	
Cantril	.3357 (14.34)	-.2687 (14.01)	-.2699 (13.87)	-.2488 (14.02)	
Life in five	-.0625 (2.28)	-.0018 (0.08)	-.1590 (7.02)	-.1824 (8.82)	
Enjoy	.0241 (5.33)	.0246 (5.33)	-.0036 (0.77)	.0044 (1.04)	
Smile	-.0260 (4.60)	-.0248 (5.29)	-.0087 (1.82)	-.0025 (0.58)	
Well rested	-.0206 (3.48)	.0087 (1.78)	-.0038 (0.76)	.0256 (5.60)	
Sad	.0189 (3.70)	.0181 (4.27)	.0227 (5.27)	.0018 (0.48)	
Anger	.0173 (3.71)	-.0032 (0.83)	.0022 (0.54)	-.0024 (0.66)	
Worry	.0411 (6.91)	.0307 (6.21)	.0600 (11.93)	.0316 (6.88)	
Pain	.0011 (0.21)	-.0046 (1.03)	.0161 (3.56)	-.0030 (0.72)	
	2019	2020	2021	2022	N
Cantril	.1330 (7.50)	.3313 (18.27)	.4126 (22.80)	.1787 (10.02)	586,158
Life in five	.2786 (13.56)	.6010 (28.67)	.6338 (30.31)	.2816 (13.63)	546,819
Enjoy	.0025 (0.60)	.0195 (4.46)	.0498 (11.46)	.0166 (3.87)	559,578
Smile	.0156 (3.41)	.0192 (4.32)	.0244 (5.51)	.0030 (0.68)	555,997
Well rested	.0130 (7.28)	.0579 (12.41)	.0318 (6.83)	.0251 (5.46)	563,259
Sad	-.0042 (1.05)	.0235 (5.85)	.0056 (1.38)	.0007 (0.18)	563,803
Anger	-.0326 (9.03)	-.0224 (6.10)	-.0183 (4.98)	-.0300 (8.30)	563,086
Worry	.0327 (7.10)	.0798 (17.00)	.0346 (7.39)	.0365 (7.95)	564,317
Pain	.0268 (6.46)	-.0040 (0.95)	.0017 (0.39)	.0164 (3.93)	565,509

Controls are country, age and age square, and female plus a full set of year dummies, a selection of which are reported above. 2008 is the excluded category. T-statistics in parentheses.

Countries are Albania; Austria; Belgium; Bulgaria; Croatia; Cyprus; Czechia; Denmark; Estonia; Finland; France; Germany; Greece; Hungary; Ireland; Italy; Latvia; Lithuania; Luxembourg; Malta; Montenegro; Netherlands; N. Macedonia; Poland; Portugal; Romania; Serbia; Slovakia; Slovenia; Spain; Sweden; Turkey and UK

Graham [60] argues that the Gallup life in five years variable tells us about *hope* and that it is associated with better future outcomes. O’Connor and Graham [61] show that on average people with higher scores on the Cantril in 5 years using rare panel data in the Gallup surveys do better over time and so do more optimistic people in a life course sense. However, while expected life satisfaction in five years drops markedly in 2009 and 2010 after the onset of the Great Recession, as in the case of Cantril, it *rises* between 2019 and 2020 with the onset of Covid, remains high in 2021, only to drop back to its 2019 level in 2022. Again, this is not what one might have expected given the size and nature of the Covid shock.

Other SWB variables also move in a somewhat unexpected manner. Enjoyment falls in 2008 relative to 2006 and 2007, but it is higher in 2020–2022 relative to the last pre-Covid year. The smile coefficient rises in 2008 relative to 2006 and 2007 and moves very little between 2019 and 2021. Being ‘well-rested’ is largely unaffected by the Great Recession but rises with the onset of COVID. Sadness in fact falls in 2008 compared to the two years either side, although it does rise in 2020 relative to 2019 and is significantly higher than it was in 2008. Anger is largely unaffected by the Great Recession and diminishes a little with the onset of Covid, albeit at levels that are significantly lower than they were in 2008. Worry is lower in 2008 than it is in 2006, 2007, 2009 and 2010. It is particularly high in 2020 but returns to 2019 levels in 2021 and 2022. Finally, although pain rises in 2009 compared to 2008, it subsides again in 2010. It is considerably higher in 2019 than it is during COVID. Taken together, these results are difficult to reconcile with the proposition that SWB metrics fall markedly–and perhaps remain low for a little while–after a major negative macro-shock.

### European Social Surveys sweeps 1–10, 2002–2020

We now move on to examining five wellbeing metrics from the biannual European Social Survey (ESS) sweeps 1–10. The first three relate to national and the last two to personal issues. The former ones fell more sharply in both 2008 and 2020 than did happiness or life satisfaction. The questions we use are defined as follows, with all variables scored 0–10.

Q5. How satisfied are you with the present state of the economy (economy)?

Q6. How satisfied are you with the national government (government)?

Q7. How satisfied are you with the way democracy works in the country (democracy)?

Q8. How satisfied are you with life as a whole (life satisfaction)?

Q9. How happy are you (happy)?

Table 10 reports the results from models that take the same form as the Gallup estimates: they are pooled regressions for 2002–2020, with 2008 as the reference year, and contain country fixed effects together with age, age squared and a female dummy variable. The first three columns present estimates for the three domain-specific satisfaction measures, namely satisfaction with the economy, government and democracy. They share two notable results. First, in each case, satisfaction plummets in 2008 relative to 2006 with the onset of the Great Recession and does not recover to its 2006 level until 2016. This seems to be a substantial and prolonged ‘hit’ from the Great Recession shock. Second, all three increased from 2014 and, in two cases (government and democracy) the coefficients are substantially bigger and more positive in 2020 after Covid than they were in 2018 before Covid. These results suggest domain specific satisfaction with society and economy in general was adversely impacted by the Great Recession, but not by Covid.

**Table 10:** Satisfaction, ESS 1–10.

	Economy	Government	Democracy	Life Satisfaction	Happiness
2002	.1480 (9.96)	.1612 (9.93)	.0725 (4.57)	-.0291 (2.06)	.0142 (1.12)
2004	.4520 (31.67)	.2082 (13.53)	.0977 (6.40)	.0357 (2.63)	.0421 (3.45)
**2006**	**.9130 (62.28)**	**.3332 (21.13)**	**.1390 (8.87)**	**.0369 (2.64)**	**.0186 (1.49)**
**2010**	**.0964 (7.08)**	**-.0679 (4.64)**	**-.0992 (6.81)**	**.0665 (5.12)**	**.0136 (1.17)**
2012	.1523 (11.02)	.0319 (2.15)	.2955 (19.99)	.1951 (14.76)	.1180 (9.95)
2014	.4791 (32.02)	.0622 (3.86)	-.0424 (2.66)	.1425 (9.96)	.1124 (8.76)
2016	.9506 (64.98)	.3768 (23.96)	.1791 (11.47)	.3577 (25.60)	.2974 (23.73)
2018	1.3828 (96.39)	.5375 (34.82)	.3479 (22.75)	.4203 (30.69)	.3587 (29.22)
2020	1.3518 (82.35)	.8586 (48.73)	.6933 (39.74)	.7130 (45.42)	.4299 (30.60)
Age	-.0384 (41.36)	-.0426 (42.51)	-.0405 (40.91)	-.0463 (52.86)	-.0271 (34.51)
Age^2^ *100	.0358 (38.69)	.0449 (45.08)	.0003 (37.91)	.0003 (43.20)	.0153 (19.63
Female	-.2030 (31.07)	-.0511 (7.25)	-.1179 (16.93)	.0188 (3.02)	.0420 (7.51)
_cons	4.9901	4.886	6.0323	7.9262	7.9579
Adjusted R^2^	0.2521	0.1227	0.1741	0.1616	0.1268
N	452,934	446,727	445,008	461,446	461,191

Satisfaction with the economy mean = 4.53

Satisfaction with government mean = 4.22

Satisfaction with democracy mean = 5.17

Life satisfaction mean = 6.88

Happiness mean = = 7.21

Equations include country dummies. The reference year is 2008. T-statistics are in parentheses.

The last two columns are qualitatively different SWB measures in that they capture individuals’ evaluations of their lives. Life satisfaction and, to a lesser extent, happiness, dipped temporarily in 2008 but both rose markedly subsequently including through Covid such that the coefficients for life satisfaction and happiness were significantly higher under COVID than they had been at any point in the preceding two decades.

### Life satisfaction Eurobarometers, 1973–2023

We now turn to data on life satisfaction taken from the Eurobarometer (EB) survey series micro data files for 1973–2023. Kelsey O’Connor has pointed out to us that the EB sample initially excluded non-natives of all countries. It was then expanded to include nationals of other EU countries. Also, the more recent Eurobarometers only cover the nationalities of EU member states [62, p. 261].

We took 141 individual Eurobarometer files and merged them together. The main question we use for comparison purposes is a 4-step life satisfaction variable coded as follows to ensure a higher number implies having higher satisfaction.

Q12. On the whole, are you very satisfied (= 4), fairly satisfied (= 3), not very satisfied (= 2) or not at all satisfied (= 1) with the life you lead?

The entire time series is presented in S2 Table by year. Part A of S3 Table reports by country life satisfaction by the eight surveys from April-May 2007 to October-November 2009, covering the major drops in output observed in the Great recession. There is a small drop in life satisfaction across these eight surveys overall as shown below in Table 11.

**Table 11:** Life satisfaction in Eurobarometer, 2007–2009.

1) 2007–2.92	#67.2–2.93
	#68.1–2.91
2) 2008–2.88	
	#69.2–2.89
	#70.1–2.87
3) 2009–2.88	
	#71.1–2.85
	#71.2–2.92
	#71.3–2.87
	#72.4–2.86

Although not inconsequential these changes are relatively small. Over the eight barometers listed above from 2007–2009 the series has a peak of 2.94 and a minimum of 2.85, a drop of .09 of a life satisfaction point.

On average the twenty surveys from 2019–2023 have a maximum of 3.07 and a minimum of 2.92 or 0.15 life satisfaction points. To put this in context, as shown in Table 12 below, the difference between the unemployed and middle management in these data is 0.52 and between the least educated and most educated, based on age left school, is 0.41 life satisfaction points.

**Table 12:** Life satisfaction in Eurobarometer, 2019–2023.

Unemployed	2.65	≤14 years	2.76
Student	2.92	15 years	2.87
Homeworker	2.92	16 years	2.97
Retired	2.99	17 years	2.91
Skilled manual worker	2.91	18 years	2.87
Unskilled manual worker	2.82	19 years	2.96
Middle management	3.21	20 years	3.10
Professional, lawyer etc.	3.17	≥21 years	3.17

Table 13 examines life satisfaction changes for Eurobarometer surveys going back to 1973. The table focuses on the periods around the Great Recession and Covid but the full series is presented in S4 Table. We report the results overall and by region where we regress life satisfaction on a set of survey dummies and, in the ‘all’ regression reported in row 1, a full set of country dummies. We provide separate results for nine Western European countries that were included in the survey series at the outset. Column 2 restricts the sample to these nine. The third column is for ten Northern and Southern European countries; mostly from 1986, with a few recent years for Iceland, Norway and Switzerland. The final column is for twenty-one Ex-Soviet bloc countries from 2004. Moldova is only included in 2023.

**Table 13:** Life satisfaction by 141 European surveys, Eurobarometers 1973–2023.

	All	Western	Southern & Northern	Eastern
Apr-May 2007	.0292 (4.01)	.0404 (4.48)	-.0678 (2.86)	.0869 (9.65)
Sep-Nov 2007	.0056 (0.77)	.0263 (2.92)	-.0878 (3.71)	.0554 (6.13)
Mar-May 2008	-.0144 (1.98)	-.0056 (0.63)	-.0848 (3.58)	.0306 (3.40)
Oct-Nov 2008	-.0372 (5.10)	-.0168 (1.87)	-.1456 (6.15)	.0194 (2.15)
Jan-Feb 2009	-.0575 (7.88)	-.0065 (0.73)	-.1669 (7.05)	-.0218 (2.42)
July-August 2020	.1204 (16.71)	.0927 (10.25)	-.0347 (1.47)	.2313 (26.76)
Aug-Sep 2020	.1165 (15.80)	.0418 (4.66)	-.0587 (2.49)	.2907 (30.82)
Oct-Nov 2020	.0389 (5.25)	-.0093 (1.00)	-.1206 (5.10)	.1764 (18.70)
Feb-March 2021	.0161 (2.29)	-.0478 (5.35)	-.1819 (7.82)	.1726 (20.42)
Mar-Apr 2021	.0405 (5.44)	-.0149 (1.59)	-.1214 (5.13)	.1846 (19.50)
Country dummies	40	8	9	20
Cons	2.8931	2.8919	2.5529	2.8527
Adjusted R^2^	0.1699	0.1263	0.1883	0.083
N	3,226,021	1,336,185	796,865	1,092,971

All equations include a female dummy. Excluded category for columns 1 & 2 is Sept-Oct 1973. For column 3 it is March-April 1981. T-statistics in parentheses.Full equation and definitions of regions in S4 Table

We make September-October 1973 the excluded category in columns 1 and 2, which we use as the base case scenario, March-April 1981 in column 3 and Oct-November 2004 in the final column.

Table 13 shows there is a small drop in life satisfaction in Western Europe with the onset of the Great Recession, and a much larger one in Southern and Northern Europe–recall that unemployment rates in Spain and Greece peaked at over 25% in this period. We also see substantial drops in satisfaction between December 2019 and February-March 2021, but life satisfaction recovers quickly in all cases.

In Table 14 we rerun the estimates in Table 13 having aggregated the data annually for all three regions and show it is much less clear that there are drops in 2007–2009, especially in Western Europe. There is a notable fall in 2020.

**Table 14:** Life satisfaction by European region by year, 1973–2023.

	Western	Southern and Northern	Eastern
**2007**	**.0441 (6.39)**	**.0367 (4.39)**	**.0447 (7.09)**
**2009**	**.0507 (8.48)**	**-.0355 (4.91)**	**-.0313 (5.72)**
2010	.0543 (8.63)	-.0467 (6.17)	-.0230 (3.92)
2018	.0960 (17.31)	.0632 (9.45)	.1696 (33.13)
**2019**	**.1277 (23.35)**	**.0943 (14.29)**	**.1998 (39.56)**
**2020**	**.0547 (8.62)**	**.0437 (5.76)**	**.2076 (35.77)**
2021	.0444 (7.64)	.0179 (2.61)	.2204 (42.22)
2022	.0748 (11.73)	.0600 (8.15)	.1983 (35.20)
2023	.0070 (5.95)	-.0052 (5.70)	.2043 (28.83)
Constant	2.8789	2.4369	2.8691
R^2^	0.1244	0.1831	0.0782
N	1,336,185	796, 865	1,092,971

Full set of dummies also included from 1974–2005 and 2011–2017 but these results are not reported. T-statistics in parentheses.

Table 15 is restricted to the seven surveys from December 2019 (#92.4) through April-May 2021. The first row presents a regression run for all countries. The remaining rows present the same estimates for separate countries. It is clear that there were major falls in satisfaction overall in these three surveys, October-November 2020 (#94.1), February to March 2021 (#94.3) and April-May 2021 (#95.2). Then life satisfaction rose in April-May 2020. A similar picture is found by country. These short-lived changes may not be picked up in annual data.

**Table 15:** Life satisfaction under Covid by survey in major countries, 2019–2021—Eurobarometers #92.3-#95.2.

	December 2019	July-Aug 2020	Aug-Sep 2020	Oct-Nov 2020	Feb-Mar 2021	Mar-Apr 2021	April-May 2021	N
All	.0367 (6.62)	.0129 (2.47)	.0110 (2.00)	-.0703 (12.61)	-.0818 (15.98)	-.0685 (12.25)	.0044 (0.86)	250,283
Austria	.0515 (1.63)	-.0835 (2.63)	-.0851 (2.68)	-.1182 (3.72)	-.2084 (6.57)	-.2080 (6.57)	-.1094 (3.44)	8,064
Belgium	-.0107 (0.39)	.0541 (1.96)	-.0364 (1.31)	-.1176 (4.29)	-.0751 (2.75)	-.0437 (1.59)	-.0108 (0.39)	8,151
Denmark	.0077 (0.29)	.0141 (0.54)	.0015 (0.06)	-.1413 (5.44)	-.2784 (10.60)	-.2726 (10.48)	-.1635 (6.32)	8,227
Finland	-.0234 (0.86)	-.1166 (4.28)	-.1653 (6.17)	-.1820 (6.69)	-.2338 (8.74)	-.2294 (8.41)	-.2104 (7.73)	8,300
France	.0789 (2.53)	.0417 (1.33)	.0420 (1.34)	-.0067 (0.21)	-.0437 (1.52)	-.0293 (0.94)	.0369 (1.18)	8,085
Germany	.0036 (0.15)	.0485 (2.13)	.0273 (1.20)	-.0660 (2.94)	.0071 (0.31)	-.0244 (1.07)	.0605 (2.65)	12,254
Greece	.1490 (4.27)	.1476 (4.224	.1904 (5.47)	.0478 (1.38)	.0460 (1.33)	.1406 (4.03)	.1870 (5.42)	8,202
Ireland	.0238 (0.83)	-.0015 (0.05)	-.1800 (6.61)	-.1527 (5.40)	-.1740 (6.21)	-.1337 (4.70)	-.1000 (3.49)	8,446
Italy	.0420 (1.40)	.0228 (0.76)	.0195 (0.65)	-.0580 (1.93)	-.1378 (4.59)	-.1235 (4.12)	.0610 (2.03)	8,172
Malta*	.0507 (1.39)	.0029 (0.08)	.0769 (2.11)	-.0328 (0.92)	-.0090 (0.26)	.0235 (0.66)	.1376 (3.83)	4,096
Netherlands	.0377 (1.41)	.0046 (0.17)	-.0420 (1.60)	-.0817 (3.06)	-.1121 (4.19)	-.0584 (2.18)	-.0282 (1.07)	8,244
Portugal	.0724 (2.68)	.0574 (2.14)	.1042 (3.91)	-.0615 (2.29)	.0224 (0.85)	.0977 (3.64)	.1543 (5.71)	8,310
Spain	.0398 (1.36)	.0613 (2.10)	-.0769 (2.65)	-.0617 (2.12)	-.1476 (5.04)	-.0855 (2.93)	-.0031 (0.11)	8,126
Sweden	.0003 (0.01)	-.0354 (1.30)	-.1759 (6.35)	-.1207 (4.40)	-.1827 (6.76)	-.2384 (8.71)	-.1484 (5.42)	8,317
UK	.0526 (1.77)	-.1300 (4.50)	-.1563 (5.31)		-.2522 (8.97)		-.1695 (5.66)	6,526

Excluded Nov-Dec 2019 = eb #92.3

All equations include a female dummy. In the case of the overall equation a full set of country dummies are also included. * = not included in the list of 25 countries examined by Easterlin and O’Connor [63].

Finally, at the end of the survey period covered by the Eurobarometers there is a third major event—the invasion of Ukraine by Russia, on Thursday, February 24, 2022. We have two surveys in our files for the subsequent period—#97.5 (June-July 2022) and #98.2 (Jan-Feb 2023). Overall, we saw no sign of a decline in life satisfaction in our sample but, as we see in Table 16 below, we did observe declines in the four countries bordering Ukraine, likely most impacted by the war.

**Table 16:** Life satisfaction in countries bordering Ukraine, Eurobarometer.

	Overall	Hungary	Poland	Romania	Slovakia
June-July 2022	2.99	2.94	3.09	2.70	2.92
January-Feb 2023	2.98	2.78	3.01	2.63	2.77

There are no obvious declines in other countries.

### Backward looking data from the European Commission surveys, 1985–2023

We now move on to another new data source from the European Commission, who also run the EB survey series, that has data available by country, year and month. We do not have the micro-data but have cell averages. There is data available from consumers on their own circumstances as well as the national economy, and both backward looking–what happened over the last twelve months–and forward looking–what is going to happen over the next year.

Not only do we have data available on expectations, but we also have backward-looking data relating to the prior twelve months on both financial situation and the general economic situation [64].

*Q13*. *How do you think the general economic situation in the country has changed over the past 12 months*? *It has*…

Got a lot better (PP)

Got a little better (P)

Stayed the same (E)

Got a little worse (M)

Got a lot worse (MM)

*Q14*.. *How has the financial situation of your household changed over the last 12 months*? *It has*

Got a lot better (PP)

Got a little better (P)

Stayed the same (E)

Got a little worse (M)

Got a lot worse (MM)

In Table 17 we regress these variables on year and country fixed effects and find that they both fall sharply in 2008 and remain low through 2012, with the declines in the general situation especially large. In terms of country effects, the lowest numbers for financial situation are found in Bulgaria, Greece and Hungary, with the highest in Denmark and Finland. In contrast in the second column relating to the general economic situation, Denmark and Serbia are highest and Greece lowest. They also fell sharply in 2020.

**Table 17:** Financial and economic situation over the last twelve months, 1986–2023.

	Financial situation	General economic situation
2007	8.3807 (7.93)	12.0631 (6.35)
2008	-.0730 (0.07)	-11.5064 (6.06)
**2009**	**-6.4067 (6.07)**	**-32.8869 (17.33)**
**2010**	**-6.3705 (6.04)**	**-17.6030 (9.28)**
**2011**	**-7.2900 (6.91)**	**-15.6182 (8.23)**
**2012**	**-8.8851 (8.46)**	**-21.4278 (11.35)**
2019	14.5605 (13.97)	13.7683 (7.35)
**2020**	**8.7953 (8.42)**	**-12.3724 (6.59)**
**2021**	**6.6106 (6.30)**	**-18.5755 (9.84)**
**2022**	**-3.3756 (3.22)**	**-25.6989 (13.61)**
**2023**	**-5.3876 (4.25)**	**-27.5381 (12.09)**
Constant	-11.8933	-29.127
Adj R^2^	0.6052	0.5101
N	10,708	10,708
Average	-12.96	-25.19

European Commission Surveys. Equations include a full set of country dummies and year dummies from 1986–2006 and 2013–2018 –not reported. 1985 is the reference year. T-statistics in parentheses

### Expectations from European Commission monthly surveys, 1985–2023

Each month the European Commission runs a series of surveys across EU countries and candidate countries of firms and consumers [65]. Here we focus on surveys of consumers who report their views on the ‘current situation’ as well as for their expectations for the year ahead. We focus on three:

a) The financial situation over the next twelve months.

b) The general economic situation over the next twelve months.

c) Unemployment expectations over the next twelve months.

These variables are calculated from individual survey responses.

Q15. How do you expect the financial position of your household to change over the next 12 months? It will…

+ + get a lot better (PP)

+ get a little better (P)

= stay the same (E)

− get a little worse (M)

− − get a lot worse (MM)

Q16. How do you expect the general economic situation in this country to develop over the next 12 mon ths? It will…

+ + get a lot better (PP)

*+ get a little better (P*)

= stay the same (E)

− get a little worse (M)

− − get a lot worse (MM)

*Q17*. *How do you expect the number of people unemployed in this country to change over the next 12 months*? *The number will*…

+ + increase sharply (PP)

+ increase slightly (P)

= remain the same (E)

− fall slightly (M)

− − fall sharply (MM)

S5 Table shows how these variables moved between 2007 and 2009 along with the unemployment rate. S6 Table plots changes by country in the movements of the unemployment expectations variable.

Based on the distribution of responses to the question an aggregate balance based on the proportions giving different answers is calculated. Hence PP+P+E+M+MM+N = 100. Balances are the difference between positive and negative responses, measured as percentage points of total answers. The score is calculated as B = (PP + 1⁄2P) − (1⁄2M + MM) which means the scores can vary between -100 and +100 [66].

**Fig 4:**
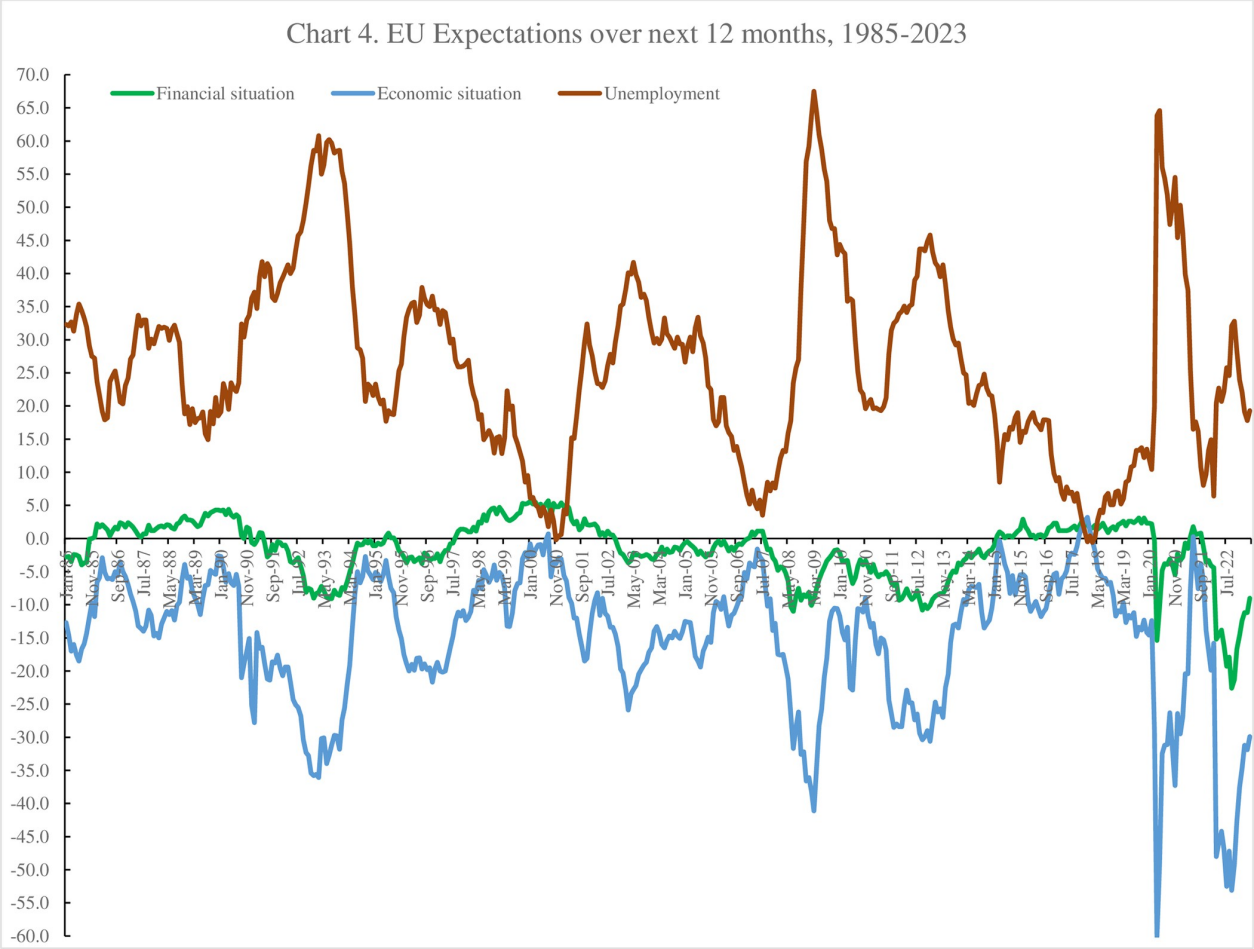
EU expectations over the next 12 months, 1985–2023.

Data are available separately for 33 countries as well as for the EU as a whole and the Eurozone. Fig 4 plots the three series from January 1985 through February 2023. The unemployment expectations variable is the mirror image of the other two series–as unemployment rises and times worsen this series rises. As the economy slows unemployment expectations rise and expectations of an individual’s financial situation and the economy as a whole fall. It is apparent that financial situation expectations relating to the individual themselves, and comparable to the life in five years in the GWP, is the least volatile of the three although it seems to track peaks and troughs. It is notable from below for the European Union as a whole that both the economic situation expectations and the unemployment expectations variables fell (rose) rapidly in 2007 and 2008 as the unemployment rate rose.

The unemployment rate started to rise in April 2008 and then rose by 0.2pp a month from September 2008. Both the general economic and unemployment expectations started to fall (rise) around the end of 2008, a year earlier.

Table 18 shows the year dummy coefficients and t-values when these three variables are regressed on year, month and country. 2008 is the reference year for the year coefficients. In column 4 we also add the overall consumer confidence variable (C. Confidence) the EU constructs as the sum of a) the financial situation over the last 12 months b) financial situation over the next twelve months c) major purchases over the next t12 months d) the economic situation over the next 12 months all summed and divided by four.

**Table 18:** Expectations over the next 12 months, by month and year, 2008–2023.

	Financial	Economic situation	Unemployment	Consumer confidence
**2007**	**8.2176 (12.61)**	**17.2159 (17.47)**	**-17.1131 (14.82)**	**9.5871 (15.20)**
**2009**	**-1.5910 (2.45)**	**-.4181 (0.43)**	**27.7577 (24.12)**	**-4.1457 (6.59)**
2010	.3319 (0.51)	8.4445 (8.59)	7.1729 (6.23)	-1.2913 (2.05)
**2019**	**12.0796 (19.12)**	**14.3042 (14.97)**	**-18.9705 (16.95)**	**10.9895 (17.97)**
**2020**	**5.3078 (8.35)**	**-2.6628 (-2.77)**	**10.6960 (9.50)**	**1.9743 (3.21)**
2021	8.4879 (13.22)	9.9564 (10.25)	-5.3383 (4.69)	6.5686 (10.57)
2022	-7.3112 (11.39)	-13.9566 (14.37)	-3.7130 (3.26)	-6.8353 (11.00)
2023	-1.6742 (1.83)	-4.6293 (-3.35)	-5.8547 (3.61)	-3.8664 (4.37)
Constant	-3.6642	-24.6635	26.1081	-13.8063
Adjusted R^2^	0.5689	0.4577	0.4911	0.5687
N	10,708	10,708	10,693	10598
Average	-3.75	-11.5	22.14	-11.73

Notes: COF - Confidence Indicator (Q1 + Q2 + Q4 + Q9) / 4. Q1. Financial situation over last 12 months. Q2. Financial situation over next 12 months. Q4. General economic situation over next 12 months. Q7. Unemployment expectations over next 12 months. Q9. Major purchases over next 12 months. Equations include 11 month and 32 country dummies. and year dummies from 1986–2006 and 2013–2018 –not reported T-statistics in parentheses.

Of note is the much larger response around 2008 and 2020 for the two macro variables compared to the micro variable regarding the respondent’s own financial situation plus the confidence aggregate. In the case of the general economic situation the coefficient for 2007 is +17 and is significantly higher than 2008, which is set to zero. The 2009 coefficient is essentially zero. Analogously the 2007 coefficient for unemployment expectations is -17 in 2007 versus +28 in 2009. Unemployment expectations also shift strongly with Covid: relative to 2008 the 2019 coefficient is -19 but it is +11 in 2020.

In Table 19 we report various unemployment rate regressions, across year, month and country. Each includes a lagged dependent variable—the 12-month lag on the unemployment rate–which has a coefficient of around .8 in all specifications. In column 1 we first include the unemployment expectations term which is positive and significant. We then experiment with the inclusion and exclusion of various expectations., all of which appear robust and operate in the anticipated fashion, predicting future unemployment.

**Table 19:** Unemployment and four expectations variables using month*country cells, Europe January 1985-March 2023.

Unempt rate_t-12_	.8337 (193.20)	.8006 (170.68)	.8392 (192.34)	.8438 (190.85)	.8416 (189.43)
Unemployment_t-12_	.0289 (38.09)	.0319 (41.13)	.0260 (29.74)		
Financial situation _t-12_		-.0503 (32.79)		-.0248 (12.27)	-.0257 (12.55)
Economic situation_t-12_	-.0309 (33.86)	-.0043 (3.44)	-.0041 (3.25)		
Industry employment_t-12_		-.0197 (15.64)			
_cons	0.7373	1.5152	1.1518	0.4008	0.3376
Adjusted R^2^	0.928	0.9249	0.9254	0.9379	0.9393
N	9,532	9,532	9,532	9,516	9,237

T-statistics in parentheses.

All equations include country, month and year dummies.

Column 2 includes all three variables (expectations regarding unemployment, the economic situation and one’s own financial situation) plus a variable capturing industry employment expectations lagged a year. This variable requires explanation. Each month the European Commission not only conducts consumer sentiment surveys but also conducts surveys amongst firms. Indeed, the Commission runs surveys across four sectors–industry; retail; services and construction (https://economy-finance.ec.europa.eu/economic-forecast-and-surveys/business-and-consumer-surveys_en). Each respondent in these surveys is asked about employment in the future–with a small variation in the question as follows. Representatives of industrial firms report employment expectations ’for the months ahead’. In contrast services, retail and construction all ask for views on employment expectations ’over the next 3 months’.

**Fig 5:**
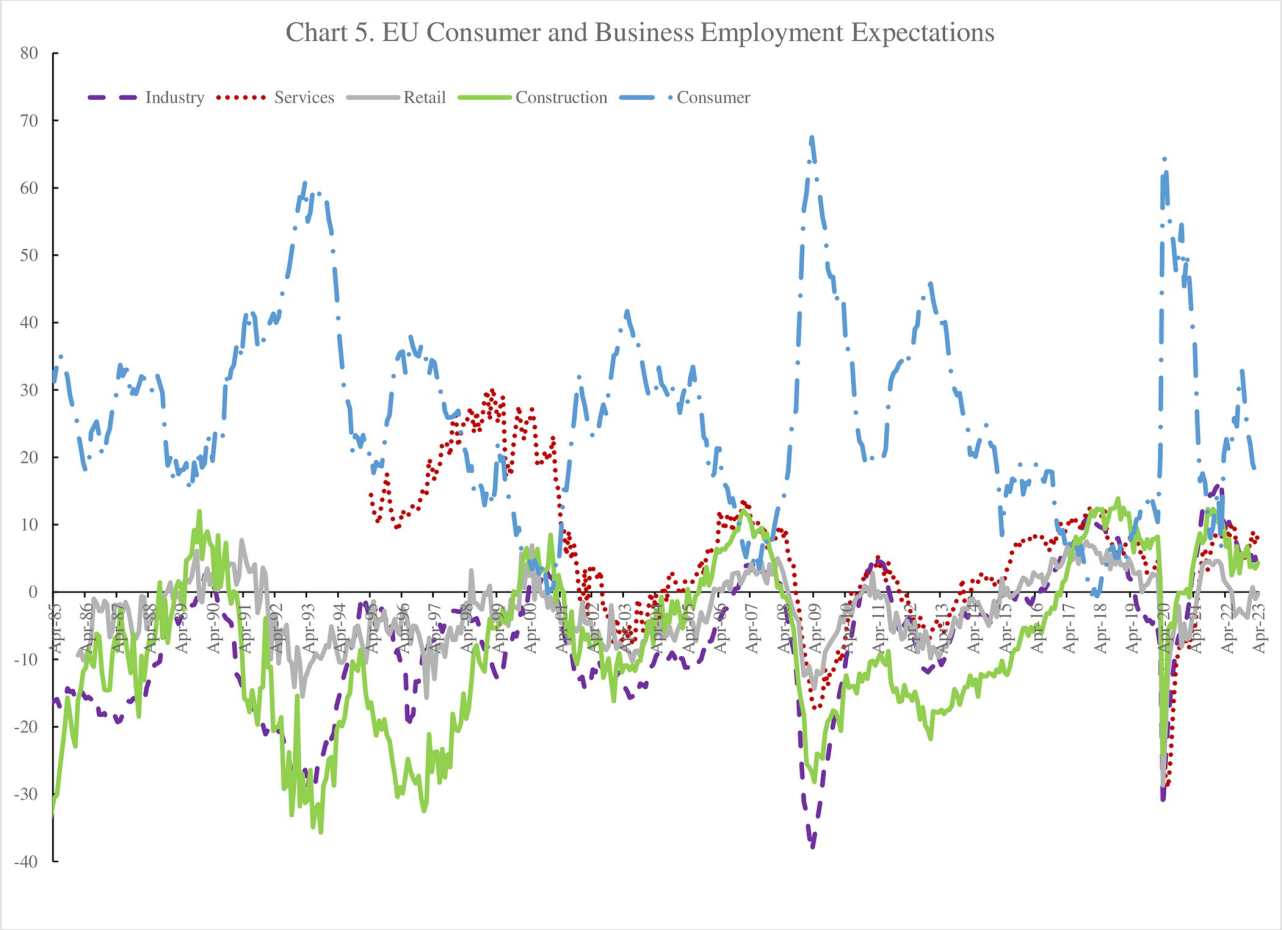
EU and consumer and business employment expectations.

As can be seen from Fig 5 they do move closely together and are the mirror image of consumers’ \unemployment expectations–employment rising is equivalent to unemployment falling. The fact that the consumer variable moves on track with the business surveys is a helpful validation. We decided to include as a control the industry fear variable lagged 12 months. This is significantly negative in column 2. Of note is how little the other coefficients change, and all remain significant with high t-values.

Table 20 repeats what was done in Table 19 but now includes the two backward looking variables which are always significantly negative. Their inclusion reduces the coefficients on the unemployment expectations variable as well as on the industry variable, but both remain highly statistically significant.

**Table 20:** Unemployment equations adding two backward looking variables, Europe January 1985-March 2023.

Unempt rate_t-12_	.7917 (187.16)	.7964 (183.03)	.7959 (185.66)	.8408 (202.02)	.7941 (187.42)
Unemployment_t-12_	.0130 (17.49)	.0255 (34.35)	.0133 (17.90		
Financial situation _t-12_		-.0034 (32.79)			
Economic situation_t-12_	-.0058 (33.86)				
Industry employment_t-12_	-.0371 (31.90)	-.0203 (17.49)			
Fin sitn last 12mths	-.0379 (21.84)	-.0387 (19.26)	-.0400 (22.77)		-.0387 (22.26))
Econ sitn last 12mths	-.0179 (20.13)	-.0219 (24.99)	-.0197 (20.96)		-.0129 (13.79)
_cons	0.7575	0.9752	0.9625	0.6662	0.3376
Adjusted R^2^	0.9437	0.9423	0.9421	0.9347	0.9455
N	9,516	9,516	9,532	9,249	9,237

T-statistics in parentheses.

All equations include country, month and year dummies.

Expectations variables appear to be highly sensitive to economic shocks and appear to have predictive power. A number of other surveys contain expectations data, including several Eurobarometers that is the subject of ongoing research. These also appear to respond sharply both during, and before the Great Recession and during the Covid years of 2020 and 2021.

## Discussion and conclusions

In this paper we examined micro-data on Europe from six micro surveys–the Gallup World Poll 2005–2023, the US BRFSS, 1993–2022, Eurobarometer 1975–2022, the daily UCL Covid Social Survey of 2020–2022, the European Social Survey 2002–2020 and the IPSOS Happiness Surveys of 2018–2023. There was evidence from four of the five micro surveys (EB, ESS, CSS, IPSOS) that both life satisfaction and happiness fell, to some extent, with these negative shocks. There was also micro-evidence from the BRFSS for the United States that despair has risen over time and increased in both the Great Recession and during the COVID pandemic and subsequently.

However, other SWB metrics captured in the GWP for Europe showed little if any evidence of a change in wellbeing with the advent of economic shocks. Wellbeing metrics such as enjoyment, smiling, sadness, anger, worry and pain, did not move as one might have expected in response to two recent major negative shocks, the Great Recession of 2008/9 and the Covid pandemic of 2020/21. For example, the probability of a respondent reporting zero on the Cantril life satisfaction scale, from 0–10, fell from 2008–2009 for Europe from 2007–2008 but rose very slightly from 2019–2020.

In part this may be because the GWP survey is collected at various points throughout the year which vary by country which means any short run, seasonal changes may be missed. For example, in 2020 it was collected in six separate months among the European sample, mostly in September, October and November. In 2019 the data were collected in eight quite different months. In 2019 Germany was sampled in June and in 2020 in September. Spain was sampled in May 2019 and September 2020.

Care has to also be taken as countries are not always present in the surveys every year, so it is an unbalanced panel. We ran Cantril regression equations (available on request) for the twelve European countries that have data available for all six years that we pooled—2007, 2008, 2019–2021. These are Belgium, Denmark, Estonia, Germany, Italy, Latvia, Lithuania, Netherlands, Spain, Sweden, Turkey, UK. The sample is restricted to these years and there is little evidence of down movements in life satisfaction in either 2008 or 2020.

People’s expectations of life in general, their financial situation and the economic and especially the employment situation in the country, all dropped markedly in the Great Recession and during Covid, but bounced back quickly, as did firms’ expectations of the economy and the labor market. The United Nations Human Development Index (HDI) did not shift much in response to negative shocks. Instead, the HDI–like life satisfaction and individuals’ expectations for themselves and their country—has been rising in the last decade reflecting overall improvements in economic and social wellbeing.

**Fig 6:**
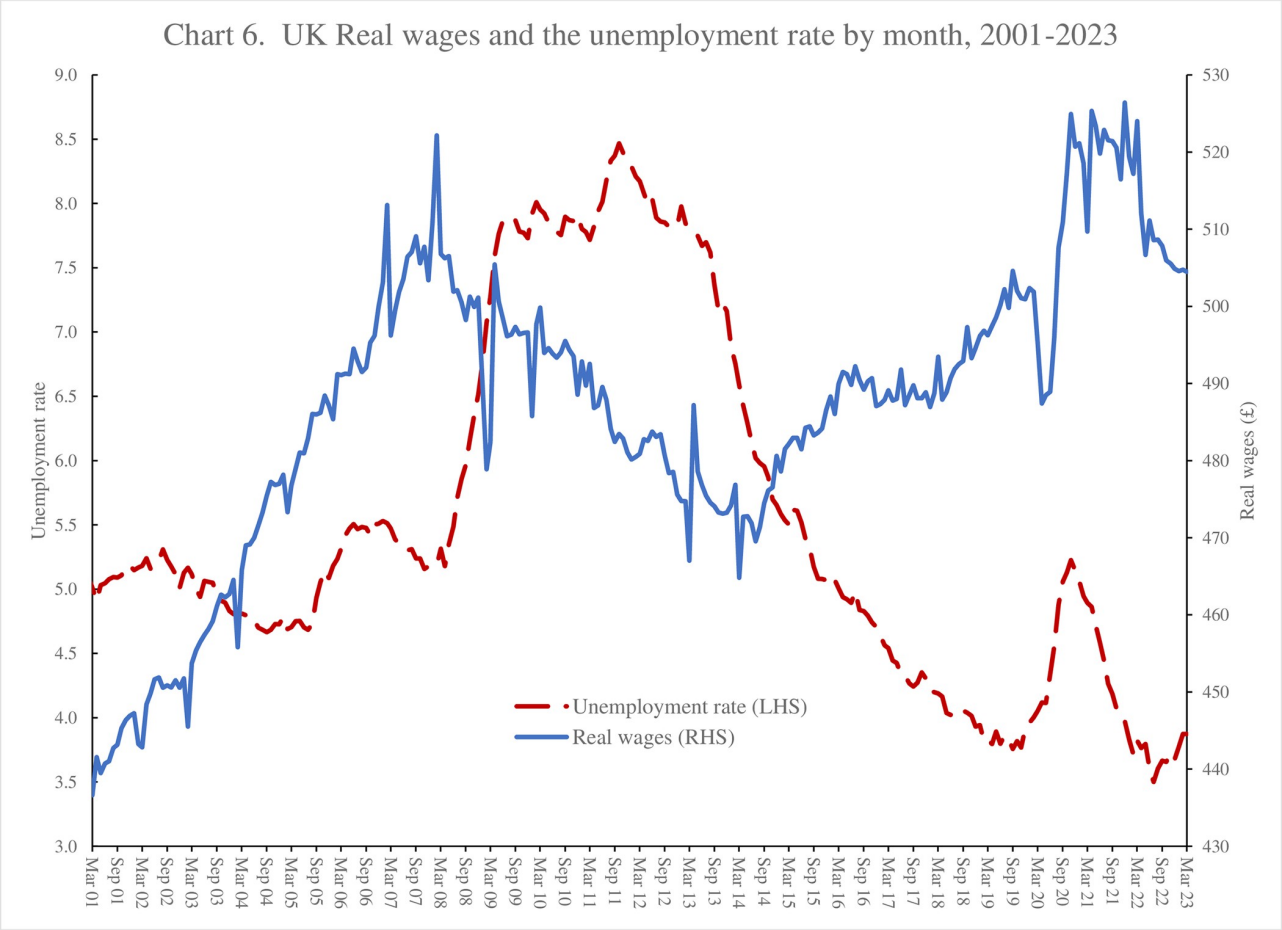
UK real wages and unemployment rate by month, 2001–2023.

One potential reason for this improvement in SWB is the growth in real earnings, apparent in many European countries, and in the OECD more generally, which dates back to around 2014. This is depicted across OECD countries in Table 21. Fig 6 for the UK shows the initial rise in the unemployment rate which then turned downwards, followed by a steady rise in the real wage which matched the rise in wellbeing. Life satisfaction for the UK from the Annual Population Survey conducted by the ONS shows a steady rise in the 10-step life satisfaction score over the years 2011–2019. The estimates by year averaged across the four quarters are 2011 = 7.40; 2012 = 7.44; 2013 = 7.48; 2014 = 7.58; 2015 = 7.65; 2016 = 7.66; 2017 = 7.68; 2018 = 7.70; 2019 = 7.68; 2020 = 7.50; 2021 = 7.50; 2022 = = 7.51.

**Table 21:** Real wage growth 2007–2021.

Location	2007	2010	2018	2021
OECD—Total	100	101	107	112
Australia	100	103	107	111
Austria	100	103	105	108
Belgium	100	101	104	106
Canada	100	103	111	114
Denmark	100	105	110	113
Finland	100	103	104	109
France	100	105	111	112
Germany	100	101	114	116
Greece	100	98	77	78
Ireland	100	113	111	114
Italy	100	102	99	97
Japan	100	98	99	101
Korea	100	102	117	120
Netherlands	100	105	104	105
New Zealand	100	101	111	119
Norway	100	105	113	119
Portugal	100	104	98	106
Spain	100	109	103	101
Sweden	100	104	114	118
Switzerland	100	102	105	108
United Kingdom	100	98	100	105
United States	100	102	109	120

Average wages are obtained by dividing the national-accounts-based total wage bill by the average number of employees in the total economy, which is then multiplied by the ratio of the average usual weekly hours per full-time employee to the average usually weekly hours for all employees. This indicator is measured in USD constant prices using 2016 base year and Purchasing Power Parities (PPPs) for private consumption of the same year. 2007 = 100. Source: https://data.oecd.org/earnwage/average-wages.htm

**Fig 7:**
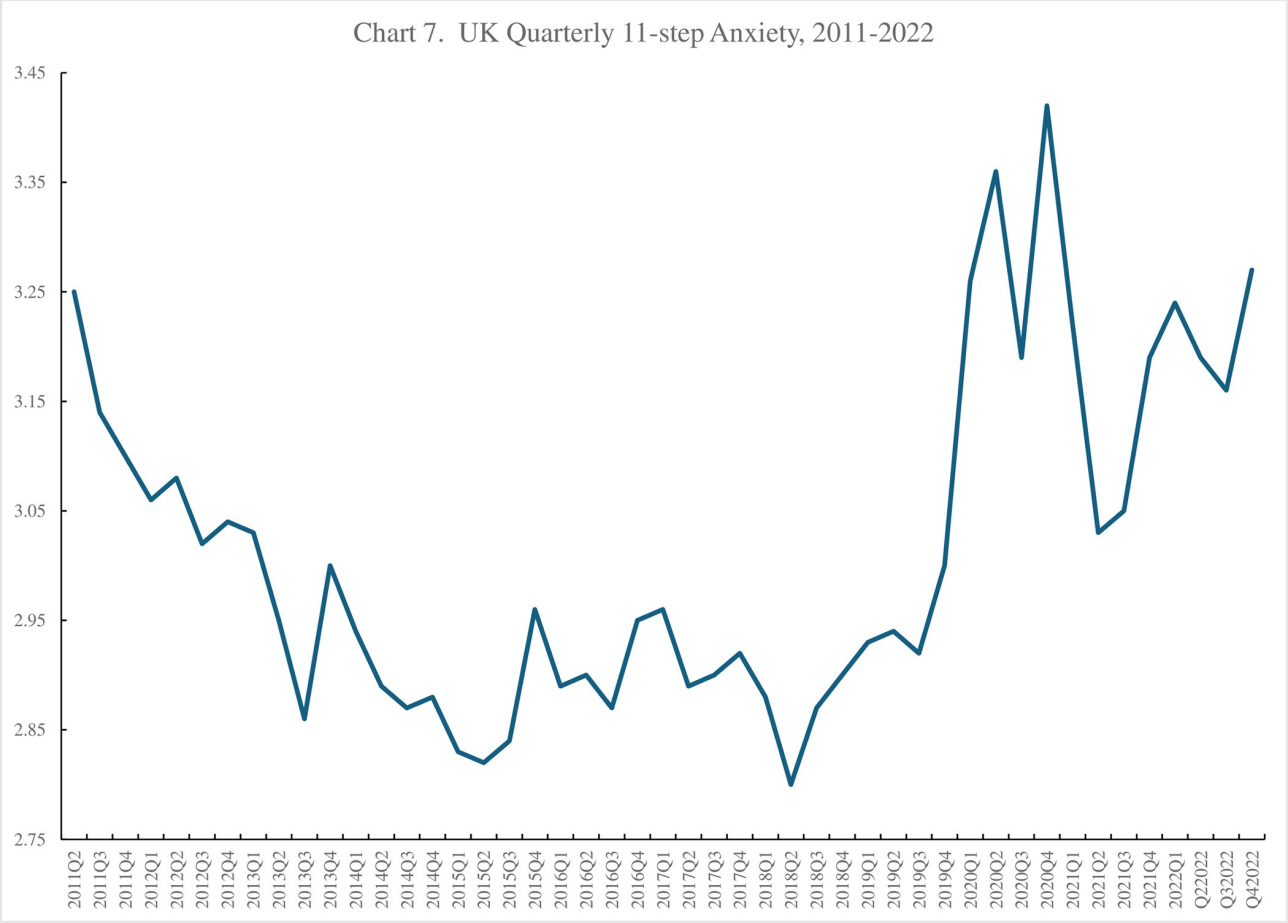
UK quarterly 11-step anxiety, 2011–2022.

However, this is unlikely to explain the secular rise in the HDI. Furthermore, we show secular trends in negative affect in Europe and despair in the United States, both of which have trended up in the last decade, suggesting that there is no simple secular rise in citizens’ wellbeing. In the UK there is evidence that alongside a rise in life satisfaction there has also been a rise in negative affect. Fig 7 plots anxiety using quarterly data from the APS survey conducted by the ONS. The series falls from 2011 to 2018 as life satisfaction rose but then started rising from 2018Q2 through 2020Q4 before falling and then rising again, from 2021Q2.

What are the implications of these analyses for the value of various SWB and expectations metrics? It seems that, with the exception of life satisfaction and domain-specific satisfaction (such as satisfaction with government and the economy in general), positive and negative affect measures do not respond as one might have anticipated to external negative shocks. The satisfaction variables are useful in this regard but, even here, movements can vary across surveys and there is substantial temporal variance which appears to be ‘noise’, suggesting one should consider the data aggregated to the year-level, comparing annual movements within countries over time to distinguish between cyclical and secular patterns.

The expectations data are somewhat different in that they move sharply in response to negative shocks but revert to previous values after relatively short periods. In this sense they are good at picking up responses to changes in economic conditions. But they have also trended upwards over time, confirming the sense that, notwithstanding concerns about the global economy after the Great Recession and Covid, there is robust evidence of secular improvement in people’s lives as indicated by their satisfaction with life, the economy, government and their expectations of the future.

We did find evidence, though, that a variety of expectations variables did move more sharply downwards in both the Great Recession and during the Covid lockdowns and were predictive. These included variables relating to the individual in both the ESS and the EB, but especially in data about the economy, in terms of the general economic situation, democracy as well as employment and unemployment. We showed that the rise in these expectations, using data on both consumers and firms from the European Commission’s Surveys of Business and Consumer Surveys from 1985–2023 was predictive of changes in the unemployment rate.

Of course, the Covid shock was different to a ‘normal’ economic shock. In "normal recessions" there is some endogeneity between economic outcomes and people’s beliefs, because through the economics of walking about, animal spirits can amplify the downturn, with a self-reinforcing loop between the deterioration of economic outcomes and wellbeing. But Covid-19 was a different kind of recession. The economy was put on halt by fiat. Expectations, confidence, animal spirits, had nothing to do with the economic downturn. Wars may be different.

The evidence as to whether the Great Recession and the COVID pandemic were happiness reducing is mixed, depending on the metrics and the study used. The weakest evidence comes from the GWP where a range of positive and negative affect metrics moved very little around the Great Recession and COVID. Life satisfaction data dips somewhat in most surveys, but the movements are small. What really moves–and moves a lot–are individuals’ expectations regarding the economy and government, as well as their satisfaction with those aspects of their lives. Why should this be? We think that what marks these expectations and domain satisfaction items out is that they are strongly evaluative, in the sense that individuals are required to reflect on macro issues–not simply their own lives–and provide an assessment.

The wellbeing metrics that underpin the UN’s Human Development Index and those contained in the Gallup World Poll data used in the World Happiness Report are crucial for mapping wellbeing and welfare within and across countries, but they are not ideal in identifying the impact of large negative shocks on welfare. For this, one needs other metrics, notably expectations data and domain specific evaluations of macro issues. This is perhaps no surprise. As Diener et al. (11, p. 277) noted some time ago: “*subjective well-being is a broad category of phenomena that includes people’s emotional responses*, *domain satisfactions*, *and global judgments of life satisfaction*. *Each of the specific constructs need to be understood in their own right*”.

## Supporting information

S1 Table Country scores and rankings from Diener and Tay (2015), Table 6. (DOCX)

S2 Table Life satisfaction by year from Eurobarometers. (DOCX)

S3 Table Life satisfaction by survey, 2019–2022 from Eurobarometers. (DOCX)

S4 Table Life satisfaction by 141 European surveys, Eurobarometers 1973–2023. (DOCX)

S5 Table EU changes in expectations and the unemployment rate 2007–2009. (DOCX)

S6 Table Unemployment expectations in 28 countries 2007–2009 and 2017–2023. (DOCX)
