# Supplementary material for: Were COVID and the Great Recession well-being reducing?
Source: PLoS One. 2024 Nov 27;19(11):e0305347. doi: 10.1371/journal.pone.0305347 (PMC11602031; doi:10.1371/journal.pone.0305347)
Supplement: S1 Table — (DOCX) [file pone.0305347.s001.docx]

Appendix Table S1

Appendix Table S1. Country scores and rankings from Diener and Tay (2015), Table 6.

Nation Material/ Physical Healthy Social SWB Equality Average

economics health environment

Iceland 94.7 90.2 84.3 90.1 82.5 77.8 86.6

Norway 94.8 86.3 82.1 93.3 81.0 78.5 86.0

Switzerland 90.5 89.7 84.3 93.0 79.1 77.6 85.7

Denmark 92.9 84.5 84.2 91.3 84.0 77.0 85.7

Luxembourg 94.8 88.3 84.3 93.2 74.4 77.9 85.5

Sweden 93.0 87.8 82.5 88.8 81.4 78.8 85.4

Singapore 89.9 93.9 90.6 88.9 73.4 74.2 85.2

Netherlands 90.9 86.5 81.1 89.7 82.6 78.0 84.8

Australia 91.7 89.5 81.2 92.3 77.8 73.8 84.4

New Zealand 88.1 89.6 85.8 89.0 77.9 73.7 84.0

Austria 86.8 88.7 82.8 88.9 79.4 76.3 83.8

Finland 86.2 86.4 82.8 91.8 79.6 76.0 83.8

United Arab Emirates 87.4 89.3 86.7 92.5 74.1 71.7 83.6

Canada 90.5 88.8 76.6 91.5 77.3 74.2 83.1

Ireland 85.2 91.3 82.1 91.3 77.6 71.3 83.1

United Kingdom 87.8 88.8 82.9 85.5 78.0 70.4 82.2

Qatar 81.9 91.3 88.6 92.2 69.4 67.7 81.8

Belgium 83.6 85.5 74.4 90.0 76.6 76.9 81.2

Germany 86.6 85.8 83.2 79.7 76.9 73.5 80.9

United States 89.8 86.3 76.0 83.5 74.3 72.5 80.4

France 82.4 90.2 72.0 87.0 72.5 76.6 80.1

Kuwait 89.9 85.7 64.7 86.8 75.7 74.7 79.6

Japan 85.5 91.1 67.8 79.3 75.2 74.3 78.9

Czech Republic 77.6 84.3 73.0 85.8 73.9 76.1 78.5

Slovenia 82.2 84.0 75.3 84.2 71.6 71.7 78.1

Bhutan 54.2 83.5 89.3 89.3 74.7 75.4 77.7

Spain 77.8 90.1 66.1 88.0 69.4 74.3 77.6

Taiwan 85.9 90.8 58.5 78.4 78.5 73.4 77.6

Hong Kong 87.9 90.9 49.5 89.4 70.5 72.7 76.8

Malaysia 65.3 82.5 76.2 87.8 77.1 70.9 76.6

Thailand 62.3 83.2 80.9 84.3 79.9 68.6 76.5

Bahrain 75.7 85.1 74.0 90.7 60.6 72.2 76.4

Costa Rica 61.2 86.8 79.8 81.7 77.7 70.4 76.3

Malta 82.1 88.8 60.1 89.3 64.6 68.3 75.6

Saudi Arabia 74.7 84.5 64.4 80.5 72.8 75.0 75.3

Uzbekistan 52.6 74.3 81.0 92.3 80.3 71.4 75.3

Uruguay 63.8 85.1 82.0 75.6 75.9 69.1 75.2

Israel 80.4 88.9 56.7 75.4 69.3 79.9 75.1

Italy 75.0 91.5 60.6 79.9 68.9 74.5 75.1

Mauritius 62.0 79.4 85.6 82.2 69.8 70.9 75.0

Slovakia 73.7 82.2 67.7 81.1 69.8 75.0 74.9

China 63.2 86.2 75.7 86.3 75.3 61.8 74.7

Portugal 74.4 84.7 75.7 78.9 67.7 67.0 74.7

Poland 72.3 80.2 68.0 81.2 73.1 72.3 74.5

South Korea 84.1 87.4 64.0 66.7 72.5 71.1 74.3

Laos 49.8 80.5 82.0 85.4 76.1 68.5 73.7

Cyprus 75.2 87.5 63.7 77.4 67.9 70.1 73.7

Venezuela 59.0 84.8 67.0 78.7 79.2 71.6 73.4

Panama 56.4 87.2 69.0 75.2 79.6 69.7 72.9

Estonia 69.5 80.4 69.1 71.3 75.0 70.3 72.6

Vietnam 57.5 83.9 70.4 85.0 69.5 69.0 72.6

Suriname 55.3 81.4 77.9 72.2 74.1 74.1 72.5

Croatia 72.0 83.1 67.6 68.4 69.4 73.2 72.3

Indonesia 51.2 82.4 72.0 79.7 77.7 69.9 72.2

Jordan 63.3 89.3 60.3 79.0 66.1 72.2 71.7

Hungary 67.1 80.6 65.4 76.7 68.2 71.3 71.5

Belarus 61.3 75.8 63.5 81.1 72.3 75.1 71.5

Latvia 66.8 79.4 67.9 69.4 71.4 72.1 71.2

Mexico 56.7 85.9 65.6 69.1 76.7 70.0 70.6

Libya 62.2 83.3 63.8 73.5 66.4 74.4 70.6

Argentina 64.7 84.9 60.8 62.5 76.8 72.5 70.4

Sri Lanka 43.7 76.8 80.6 82.7 67.0 70.4 70.2

Trinidad & Tobago 59.4 81.0 64.8 72.5 77.5 65.9 70.2

Belize 60.5 84.9 59.1 69.7 71.2 75.2 70.1

Brazil 62.9 83.2 65.7 62.5 75.7 68.3 69.7

Myanmar 39.9 77.2 84.4 77.3 74.6 63.4 69.5

Kazakhstan 57.0 76.8 52.7 79.7 77.2 72.7 69.4

Montenegro 67.2 82.6 61.2 69.6 65.1 69.9 69.3

Chile 63.5 84.9 61.8 66.1 73.1 66.1 69.2

Lithuania 65.3 80.5 58.3 63.8 70.9 72.7 68.6

Tunisia 61.4 82.5 54.9 76.1 64.6 71.7 68.5

Paraguay 55.0 83.2 68.4 60.0 80.3 64.3 68.5

Jamaica 57.4 83.7 67.9 68.7 74.1 59.5 68.5

Algeria 63.6 83.3 59.5 69.1 66.3 69.3 68.5

Greece 69.3 89.1 54.5 61.6 64.5 72.1 68.5

Puerto Rico 64.0 82.5 51.8 72.3 75.9 62.8 68.2

Kosovo 64.8 82.1 52.6 66.4 74.1 67.0 67.8

Guyana 58.4 76.9 57.7 71.9 66.9 74.7 67.8

Colombia 56.3 84.6 66.1 61.2 72.4 65.5 67.7

Kyrgyzstan 48.7 76.2 64.5 71.3 76.0 69.1 67.7

Macedonia 65.6 83.5 57.2 67.9 63.4 67.9 67.6

Tajikistan 47.7 75.7 67.0 75.5 70.1 69.2 67.5

Djibouti 48.4 73.9 62.5 77.2 74.4 68.0 67.4

Bangladesh 43.7 76.8 79.0 66.8 70.3 67.6 67.4

Ecuador 52.8 84.0 62.0 64.3 71.9 68.5 67.3

Cambodia 32.5 72.5 83.6 86.7 65.7 62.1 67.2

Nicaragua 47.0 82.4 72.1 67.5 71.4 62.1 67.1

Morocco 57.9 81.8 64.0 67.7 67.5 61.6 66.7

Guatemala 51.2 83.3 65.1 60.7 74.1 65.8 66.7

Bulgaria 63.4 79.6 51.8 69.6 68.2 66.7 66.6

Nepal 46.6 76.2 75.3 59.9 73.5 66.9 66.4

Philippines 36.2 76.5 81.9 81.7 59.2 61.7 66.2

El Salvador 49.2 84.0 63.2 60.1 72.2 67.9 66.1

India 47.2 77.2 69.3 67.1 68.0 67.1 66.0

Bolivia 50.2 76.7 68.7 63.2 66.2 69.3 65.7

Mongolia 51.2 75.9 47.8 72.8 75.8 69.9 65.6

Ethiopia 33.5 76.5 74.5 71.4 72.2 65.2 65.5

South Africa 47.6 70.3 65.1 75.8 73.7 58.8 65.2

Azerbaijan 42.5 78.7 56.8 72.2 69.0 71.4 65.1

Dominican Republic 48.3 83.3 64.8 69.5 68.1 56.4 65.1

Botswana 39.0 64.9 70.6 85.6 71.6 57.7 64.9

Honduras 43.3 82.9 66.7 63.4 72.9 60.0 64.9

Iran 59.5 82.9 64.9 68.0 53.5 60.2 64.8

Bosnia and Herzegovina 64.9 81.1 55.9 54.7 65.2 66.7 64.7

Albania 58.2 84.2 46.2 67.9 65.6 65.5 64.6

Rwanda 28.3 74.3 80.0 81.6 71.3 51.6 64.5

Romania 59.8 79.2 55.0 61.3 65.6 65.4 64.4

Russia 62.6 74.4 39.4 61.2 75.7 71.3 64.1

Serbia 64.9 80.3 47.1 59.9 62.9 68.9 64.0

Turkey 58.3 83.2 56.9 53.3 57.1 71.5 63.4

Ghana 37.5 70.9 65.7 74.3 69.9 61.2 63.2

Senegal 41.2 75.7 51.6 68.5 73.0 69.3 63.2

Moldova 54.1 75.9 49.1 60.6 70.4 69.1 63.2

Swaziland 46.6 61.7 65.2 73.4 71.1 60.7 63.1

Peru 49.2 82.1 55.7 54.9 67.2 67.3 62.7

Mauritania 40.9 73.9 51.2 65.8 74.9 69.7 62.7

Georgia 45.5 74.7 62.7 65.5 65.9 62.0 62.7

Syria 56.1 87.2 52.4 69.7 50.9 59.8 62.7

Lebanon 67.0 87.0 38.3 56.2 59.2 68.1 62.6

Kenya 31.4 75.5 62.4 70.4 73.2 62.3 62.5

Niger 22.5 71.4 68.1 72.0 74.8 66.0 62.4

Egypt 50.6 80.5 53.1 62.2 58.7 66.4 61.9

Mali 34.8 70.0 56.4 68.9 77.7 63.3 61.9

Mozambique 40.3 69.1 64.6 64.6 69.3 62.7 61.8

Sudan 43.7 73.6 54.5 67.7 66.2 64.2 61.7

Armenia 52.0 76.6 53.0 63.1 59.3 65.3 61.5

Zimbabwe 36.2 69.6 62.5 69.4 70.8 59.2 61.3

Malawi 25.6 64.9 74.9 72.3 72.0 55.6 60.9

Pakistan 45.5 72.5 56.3 58.9 65.4 66.1 60.8

Ukraine 56.2 73.2 34.0 55.6 73.0 70.6 60.4

Ivory Coast 36.8 69.7 54.4 66.9 71.1 61.9 60.1

Zambia 31.8 68.6 60.1 67.9 72.1 59.4 60.0

Palestinian Territories 56.1 82.9 47.1 49.6 57.2 66.8 60.0

Burkina Faso 28.0 70.2 59.9 66.1 69.0 63.8 59.5

Nigeria 38.5 72.0 50.2 63.4 68.8 63.9 59.5

Cameroon 33.0 67.2 59.0 66.6 68.1 62.8 59.4

Afghanistan 34.2 70.7 62.2 54.6 66.9 66.9 59.3

Yemen 40.8 75.8 50.4 58.0 63.0 65.7 59.0

Uganda 27.6 67.7 61.5 73.4 65.3 58.0 58.9

Comoros 33.0 70.8 57.2 51.2 72.9 68.1 58.9

Gabon 38.5 70.5 53.3 65.9 65.7 58.8 58.8

Madagascar 28.1 77.0 60.9 52.3 68.0 66.2 58.8

Tanzania 29.3 72.0 52.0 66.0 69.5 62.4 58.6

Benin 25.8 71.3 58.7 61.6 68.5 62.8 58.1

Angola 35.1 69.0 49.8 57.7 65.7 67.2 57.4

Iraq 55.7 77.5 35.3 49.7 50.0 72.8 56.8

Congo Brazzaville 32.4 70.1 53.8 58.5 66.0 57.9 56.5

Lesotho 28.8 63.8 42.6 71.4 75.6 52.6 55.8

Guinea 22.0 69.4 52.5 57.1 68.9 62.7 55.4

Congo (Kinshasa) 26.1 72.0 44.7 49.2 69.8 66.5 54.7

Central African Republic 14.7 65.6 63.0 53.4 68.7 61.0 54.4

Burundi 18.7 66.1 62.7 43.6 70.6 62.3 54.0

Liberia 17.4 70.5 46.9 60.5 62.3 59.0 52.8

Sierra Leone 22.7 61.3 48.9 59.9 56.5 59.3 51.4

Togo 25.1 67.8 47.9 48.3 59.5 58.8 51.2

Chad 23.2 63.8 43.3 49.5 66.9 58.7 50.9

Haiti 31.6 71.5 40.0 37.2 61.5 62.3 50.7

*Note:* Category scores for each well-being component were based on indicators that were administered to more than 160 nations. All negatively worded items were reversed-scored (R). “Economics/Material”: Annual household income, Internet, Television, Shelter (R), Food (R); “Physical Health”: Health Problems (R), Life Expectancy; “Environment”: Environment preserved, Quality water, Quality air; “Social”: Support, Freedom, Children Respected, Good place for Immigrants; “SWB”: Life satisfaction, Enjoy, Anger (R), Sad (R), Stress (R); “Equality”: Income GINI, LS GINI.
