# Supplementary material for: Were COVID and the Great Recession well-being reducing?
Source: PLoS One. 2024 Nov 27;19(11):e0305347. doi: 10.1371/journal.pone.0305347 (PMC11602031; doi:10.1371/journal.pone.0305347)
Supplement: S2 Table — (DOCX) [file pone.0305347.s002.docx]

Appendix Table S2. Life satisfaction by year from Eurobarometers

France Belgium Netherlands Germany Italy Luxembourg Denmark Ireland UK Greece Spain Portugal

1973 2.89 3.34 3.34 2.97 2.67 2.67 3.45 3.42 3.15

1975 2.85 3.31 3.25 2.92 2.59 2.59 3.48 3.20 3.18

1976 2.83 3.20 3.27 2.95 2.52 2.52 3.39 3.24 3.07

1977 2.71 3.28 3.29 3.02 2.56 2.56 3.47 3.21 3.06

1978 2.77 3.29 3.36 3.05 2.61 2.61 3.46 3.27 3.15

1979 2.75 3.34 3.37 3.05 2.60 2.60 3.54 3.23 3.17

1980 2.71 3.32 3.42 3.11 2.57 2.57 3.45 3.15 3.10

1981 2.73 3.20 3.43 3.02 2.65 2.65 3.50 3.17 3.17

1982 2.85 3.15 3.38 2.98 2.76 2.76 3.53 3.21 3.14 2.64

1983 2.78 3.01 3.35 2.99 2.65 2.65 3.51 3.14 3.16 2.62

1984 2.78 2.96 3.32 2.99 2.64 2.64 3.51 3.06 3.12 2.69

1985 2.83 3.00 3.36 3.02 2.74 2.74 3.57 3.10 3.16 2.70

1986 2.77 2.96 3.30 2.98 2.70 2.70 3.54 3.07 3.14 2.66 2.95 2.50

1987 2.80 2.99 3.29 3.00 2.72 2.72 3.49 2.95 3.14 2.67 2.95 2.69

1988 2.93 3.12 3.40 3.09 2.82 2.82 3.58 3.02 3.18 2.72 3.02 2.71

1989 2.92 3.10 3.44 3.10 2.86 2.86 3.53 3.06 3.20 2.79 2.99 2.65

1990 2.88 3.15 3.40 3.10 2.88 2.88 3.55 3.17 3.17 2.70 2.98 2.72

1991 2.97 3.28 3.49 2.95 2.99 2.99 3.61 3.23 3.16 2.69 3.07 2.78

1992 2.83 3.15 3.41 2.90 2.88 2.88 3.59 3.20 3.16 2.51 2.89 2.76

1993 2.79 3.11 3.42 2.91 2.87 2.87 3.58 3.15 3.12 2.43 2.83 2.67

1994 2.78 3.12 3.40 2.90 2.84 2.84 3.61 3.16 3.18 2.50 2.77 2.64

1995 2.85 3.02 3.31 2.94 2.85 2.85 3.56 3.27 3.19 2.46 2.82 2.68

1996 2.79 3.11 3.41 2.93 2.88 2.88 3.62 3.21 3.13 2.44 2.83 2.56

1997 2.83 3.09 3.22 2.87 2.88 2.88 3.59 3.16 3.12 2.93 2.95 2.83

1998 2.76 2.94 3.37 2.73 2.81 2.81 3.59 3.34 3.16 2.71 2.92 2.57

1999 2.91 3.04 3.37 2.91 2.91 2.91 3.66 3.25 3.16 2.72 2.97 2.65

2000 2.91 3.01 3.31 2.82 2.81 2.81 3.56 3.24 3.14 2.61 2.99 2.60

2001 2.95 3.08 3.41 2.97 2.92 2.92 3.60 3.26 3.21 2.66 3.06 2.71

2002 2.84 2.96 3.30 2.84 2.91 2.91 3.55 3.16 3.17 2.56 2.97 2.48

2003 2.85 3.04 3.29 2.76 2.86 2.86 3.57 3.16 3.19 2.67 3.02 2.50

2004 2.95 3.25 3.33 3.16 3.00 3.00 3.57 3.36 3.32 2.78 3.14 2.64

2005 2.96 3.17 3.41 2.93 2.83 2.83 3.62 3.29 3.21 2.67 3.03 2.48

2006 2.99 3.19 3.39 2.90 2.87 2.87 3.61 3.28 3.21 2.71 3.10 2.50

2007 2.96 3.18 3.44 2.97 2.79 2.79 3.63 3.23 3.22 2.68 3.06 2.52

2008 2.90 3.12 3.47 2.94 2.62 2.62 3.61 3.22 3.19 2.57 2.99 2.41

2009 2.96 3.17 3.45 2.99 2.69 2.69 3.67 3.25 3.29 2.41 2.87 2.42

2010 2.98 3.16 3.43 3.01 2.73 2.73 3.65 3.23 3.30 2.32 2.91 2.34

2011 2.92 3.14 3.45 2.96 2.73 2.73 3.54 3.13 3.28 2.35 2.82 2.53

2012 3.07 3.19 3.39 3.20 2.72 2.72 3.60 3.15 3.31 2.39 2.95 2.38

2013 2.95 3.20 3.42 3.12 2.59 2.59 3.68 3.11 3.30 2.16 2.84 2.22

2014 2.95 3.16 3.44 3.12 2.60 2.60 3.69 3.27 3.32 2.22 2.89 2.39

2015 3.04 3.20 3.49 3.16 2.69 2.69 3.71 3.34 3.40 2.32 2.97 2.55

2016 3.01 3.17 3.49 3.14 2.68 2.68 3.70 3.38 3.42 2.23 2.99 2.65

2017 3.01 3.14 3.49 3.19 2.72 2.72 3.69 3.39 3.40 2.34 3.03 2.74

2018 2.97 3.07 3.49 3.16 2.71 2.71 3.68 3.38 3.35 2.36 3.04 2.70

2019 3.01 3.14 3.52 3.21 2.73 2.73 3.71 3.36 3.37 2.46 3.11 2.73

2020 2.99 3.09 3.47 3.21 2.70 2.70 3.64 3.19 3.19 2.55 3.06 2.74

2021 2.97 3.12 3.45 3.19 2.70 2.70 3.51 3.25 3.14 2.57 2.97 2.85

2022 2.95 3.05 3.44 3.17 2.78 2.78 3.60 3.45 3.19 2.57 3.11 2.85

2023 2.96 2.98 3.49 3.07 2.82 2.82 3.69 3.41 3.20 2.59 3.08 2.79

Finland Sweden Austria Cyprus Czechia Estonia Hungary Latvia Lithuania Malta Poland Slovakia

1996 3.14 3.36 3.24

1997 3.15 3.26 3.26

1998 3.17 3.39 3.06

1999 3.16 3.34 3.20

2000 3.11 3.31 3.06

2001 3.12 3.34 3.16

2002 3.15 3.31 3.13

2003 3.15 3.29 3.08

2004 3.33 3.42 3.14 3.20 2.89 2.74 2.50 2.57 2.59 3.19 2.92 2.66

2005 3.27 3.42 3.03 3.08 2.94 2.70 2.49 2.57 2.53 3.07 2.74 2.59

2006 3.25 3.43 3.05 3.12 2.92 2.79 2.42 2.62 2.60 3.01 2.80 2.71

2007 3.26 3.41 3.05 3.09 2.92 2.83 2.40 2.66 2.66 3.05 2.85 2.75

2008 3.27 3.45 2.98 3.12 2.90 2.80 2.33 2.62 2.64 3.10 2.80 2.71

2009 3.31 3.45 3.01 3.13 2.90 2.75 2.30 2.50 2.55 2.98 2.83 2.76

2010 3.28 3.44 3.07 3.12 2.88 2.76 2.42 2.58 2.51 2.98 2.89 2.86

2011 3.25 3.42 2.98 3.03 2.86 2.70 2.40 2.55 2.54 3.00 2.80 2.78

2012 3.25 3.52 3.08 3.16 2.93 2.79 2.50 2.82 2.84 3.04 2.96 2.83

2013 3.28 3.46 3.06 2.88 2.90 2.72 2.42 2.72 2.72 3.17 2.85 2.69

2014 3.29 3.44 3.18 3.03 2.93 2.81 2.58 2.76 2.74 3.25 2.91 2.77

2015 3.34 3.48 3.17 3.07 2.99 2.86 2.66 2.83 2.83 3.28 2.97 2.80

2016 3.34 3.46 3.19 3.11 2.99 2.90 2.67 2.84 2.78 3.26 2.97 2.88

2017 3.33 3.46 3.27 3.15 3.00 2.89 2.71 2.82 2.79 3.23 3.00 2.88

2018 3.30 3.43 3.25 3.09 3.00 2.92 2.74 2.84 2.80 3.19 3.04 2.89

2019 3.31 3.45 3.27 3.14 3.08 2.94 2.81 2.89 2.87 3.11 3.01 2.92

2020 3.15 3.33 3.14 3.15 3.06 2.88 2.87 2.94 2.91 3.10 3.04 2.82

2021 3.10 3.26 3.10 3.19 3.13 2.91 2.86 2.81 2.84 3.10 3.02 2.86

2022 3.21 3.34 3.11 3.02 3.09 2.96 2.90 2.85 2.89 3.29 3.06 2.83

2023 3.34 3.39 3.08 3.02 3.09 2.96 2.78 2.90 2.91 3.37 3.01 2.77

----

Slovenia Bulgaria Romania Turkey Croatia TCC N Macedonia Montenegro Serbia Albania

2004 3.17 2.18 2.46 2.85 2.78 3.08

2005 3.11 2.04 2.34 2.89 2.74 3.01

2006 3.12 2.06 2.35 2.86 2.80 3.02

2007 3.12 2.14 2.42 2.93 2.81 2.86 2.57

2008 3.08 2.21 2.45 2.67 2.79 2.62 2.56

2009 3.05 2.20 2.40 2.59 2.78 2.56 2.56

2010 3.05 2.17 2.17 2.72 2.80 2.67 2.49

2011 2.97 2.24 2.33 2.67 2.73 2.61 2.52 2.54

2012 3.05 2.47 2.62 2.73 2.72 2.43 2.45 2.46 2.18 2.62

2013 3.01 2.13 2.34 2.73 2.81 2.37 2.54 2.50 2.24 2.67

2014 3.05 2.26 2.51 2.78 2.82 2.42 2.62 2.70 2.45 2.56

2015 3.10 2.36 2.63 2.67 2.89 2.50 2.54 2.62 2.38 2.66

2016 3.16 2.42 2.70 2.75 2.89 2.50 2.54 2.60 2.38 2.65

2017 3.20 2.47 2.69 2.86 2.85 2.74 2.63 2.76 2.44 2.52

2018 3.17 2.45 2.60 2.85 2.82 2.74 2.60 2.82 2.47 2.36

2019 3.19 2.45 2.57 2.81 2.94 2.78 2.58 2.78 2.52 2.58

2020 3.14 2.43 2.72 2.69 2.94 2.76 2.83 2.75 2.54 2.54

2021 3.07 2.60 2.81 2.62 2.93 2.90 2.85 2.78 2.68 2.70

2022 3.12 2.52 2.66 2.52 2.98 2.80 2.58 2.68 2.67

2023 3.17 2.58 2.63 2.55 2.99 2.97 2.60 2.82 2.63

----

Norway Switzerland Iceland Bosnia/Herz Moldova

1992 3.39

1993 3.38

1994 3.39

1995 3.41

1996 3.35

2001 3.42

2002 3.42

2010 3.59

2011 3.54

2012 3.56

2021 3.19 3.34 3.55 3.08

2022 3.21 3.41 3.54 3.04

2023 3.13 3.29 3.32 3.02 2.52
