# Supplementary material for: Were COVID and the Great Recession well-being reducing?
Source: PLoS One. 2024 Nov 27;19(11):e0305347. doi: 10.1371/journal.pone.0305347 (PMC11602031; doi:10.1371/journal.pone.0305347)
Supplement: S3 Table — (DOCX) [file pone.0305347.s003.docx]

Appendix Table S3. Life satisfaction by survey, 2019-2022 from Eurobarometers

**a) Great Recession, 2007-2009**

#67.2 Apr-May 2007

#68.1 Sep-Nov 2007

#69.2 Mar-May 2008

#70.1 Oct-Nov 2008

#71.1 Jan-Feb 2009

#71.2 May-Jun 2009

#71.3 Jun-Jul 2009

#72.4 Oct-Nov 2009

France Belgium Netherlands Germany Italy Luxembourg Denmark Ireland UK Greece Spain Portugal

#67.2 2.95 3.18 3.45 3.02 2.80 3.38 3.60 3.25 3.22 2.68 3.05 2.57

#68.1 2.97 3.18 3.44 2.93 2.79 3.39 3.65 3.21 3.22 2.68 3.07 2.47

#69.2 2.90 3.11 3.45 2.92 2.62 3.39 3.61 3.27 3.20 2.67 3.02 2.46

#70.1 2.89 3.12 3.49 2.95 2.61 3.30 3.61 3.17 3.19 2.48 2.97 2.36

#71.1 2.86 3.12 3.44 2.94 2.56 3.37 3.64 3.28 3.23 2.42 2.91 2.31

#71.2 3.01 3.24 3.44 3.04 2.74 3.36 3.71 3.30 3.32 2.39 2.93 2.53

#71.3 2.99 3.21 3.48 2.99 2.72 3.35 3.69 3.26 3.27 2.29 2.85 2.40

#72.4 2.95 3.11 3.46 3.01 2.73 3.36 3.66 3.14 3.33 2.54 2.79 2.44

Finland Sweden Austria Cyprus Czechia Estonia Hungary Latvia Lithuania Malta Poland Slovakia

#67.2 3.27 3.44 3.02 3.12 2.92 2.85 2.41 2.64 2.69 3.08 2.85 2.76

#68.1 3.25 3.38 3.07 3.05 2.91 2.80 2.38 2.68 2.63 3.02 2.85 2.74

#69.2 3.27 3.45 3.00 3.12 2.90 2.81 2.35 2.62 2.64 3.14 2.80 2.68

#70.1 3.27 3.45 2.96 3.12 2.90 2.79 2.31 2.61 2.64 3.05 2.80 2.74

#711 3.29 3.47 2.93 3.13 2.86 2.74 2.31 2.43 2.41 3.05 2.76 2.74

#71.2 3.36 3.47 3.05 3.15 2.94 2.77 2.38 2.59 2.64 3.03 2.91 2.75

#71.3 3.30 3.44 3.00 3.16 2.90 2.75 2.29 2.44 2.60 3.05 2.88 2.73

#72.4 3.30 3.42 3.06 3.07 2.90 2.74 2.24 2.54 2.54 2.80 2.79 2.82

Slovenia Bulgaria Romania Croatia TCyprus Turkey N Macedonia

#67.2 3.14 2.14 2.44 2.82 2.91 2.99 2.60

#68.1 3.10 2.15 2.39 2.81 2.82 2.87 2.54

#69.2 3.09 2.22 2.48 2.79 2.74 2.60 2.58

#70.1 3.06 2.19 2.42 2.79 2.49 2.74

#71.1 3.03 2.18 2.46 2.87 2.66 2.58 2.51

#71.2 3.07 2.21 2.47 2.79 2.57 2.54

#71.3 3.05 2.24 2.35 2.76 2.53 2.63 2.60

#72.4 3.04 2.16 2.34 2.71 2.50 2.56 2.58

**b) Covid, 2019-2021**

#92.3 November-December 2019

#92.4 December 2019

#93.1 July-August 2020

#93.2 August-September 2020

#94.1 October-November 2020

#94.3 February-March 2021

#95.1 March-April 2021

#95.2 April-May 2021

France Belgium Netherlands Germany Italy Luxembourg Denmark Ireland UK Greece Spain Portugal

#92.3 2.96 3.13 3.51 3.21 2.71 3.35 3.68 3.31 3.34 2.42 3.09 2.71

#92.4 3.04 3.11 3.55 3.21 2.75 3.32 3.69 3.33 3.39 2.57 3.13 2.78

#93.1 3.00 3.18 3.51 3.25 2.73 3.26 3.70 3.31 3.21 2.57 3.15 2.77

#93.2 3.00 3.09 3.47 3.23 2.73 3.16 3.68 3.13 3.18 2.61 3.01 2.81

#94.1 2.96 3.01 3.43 3.14 2.65 3.11 3.54 3.16 2.47 3.02 2.65

#94.3 2.91 3.05 3.40 3.21 2.57 3.04 3.40 3.14 3.09 2.47 2.94 2.73

#95.1 2.93 3.08 3.45 3.18 2.58 3.11 3.41 3.18 2.57 3.00 2.81

#95.2 3.00 3.11 3.48 3.27 2.77 3.16 3.51 3.21 3.17 2.61 3.08 2.86

Finland Sweden Austria Cyprus Czechia Estonia Hungary Latvia Lithuania Malta Poland Slovakia

#92.3 3.31 3.43 3.23 3.17 3.14 2.95 2.77 2.89 2.80 3.09 2.97 2.87

#92.4 3.28 3.43 3.28 3.21 3.15 2.93 2.84 2.86 2.85 3.14 2.98 2.95

#93.1 3.19 3.40 3.15 3.17 3.16 2.93 2.93 3.00 2.98 3.09 3.05 2.85

#93.2 3.14 3.26 3.15 3.23 3.09 2.84 2.85 2.97 2.97 3.17 3.06 2.84

#94.1 3.12 3.32 3.11 3.06 2.93 2.88 2.81 2.85 2.80 3.06 3.02 2.76

#94.3 3.07 3.25 3.02 2.94 3.09 2.82 2.72 2.72 2.77 3.08 2.98 2.74

#95.1 3.07 3.20 3.02 3.04 3.08 2.86 2.75 2.71 2.77 3.11 3.01 2.81

#95.2 3.09 3.29 3.12 3.22 3.16 2.92 2.82 2.73 2.81 3.23 3.06 2.85

Slovenia Bulgaria Romania Croatia TCyprus Turkey N Macedonia Montenegro Serbia Albania Bosnia Kosovo

#92.3 3.15 2.48 2.65 2.94 2.79 2.84 2.54 2.80 2.57 2.51

#92.4 3.20 2.43 2.65 3.00

#93.1 3.21 2.41 2.69 3.00 2.76 2.69 2.68 2.75 2.54 2.36

#93.2 3.21 2.42 2.85 2.99

#94.1 3.00 2.47 2.63 2.89

#94.3 2.93 2.50 2.75 2.86 2.78 2.68 2.76 2.70 2.61 2.60 2.80 3.02

#95.1 3.05 2.47 2.66 2.95

#95.2 3.12 2.61 2.76 3.01 2.67 2.84 2.85 2.70 2.55 2.84 3.12
