# Supplementary material for: Were COVID and the Great Recession well-being reducing?
Source: PLoS One. 2024 Nov 27;19(11):e0305347. doi: 10.1371/journal.pone.0305347 (PMC11602031; doi:10.1371/journal.pone.0305347)
Supplement: S4 Table — (DOCX) [file pone.0305347.s004.docx]

Appendix Table S4. Life satisfaction by 141 European surveys, Eurobarometers 1973-2023

All Western Southern & Northern Eastern

May 1975 -.0378 (4.00) -.0378 (4.19)

Oct 1975 -.0857 (8.95) -.0856 (9.36)

May-Jun 1976 -.0826 (8.52) -.0826 (8.90)

Nov-Dec 1976 -.0763 (8.03) -.0763 (8.40)

Apr-May 1977 -.0744 (7.79) -.0743 (8.14)

Oct-Nov 1977 -.0184 (1.92) -.0184 (2.00)

May-Jun 1978 -.0322 (3.39) -.0322 (3.54)

Oct-Nov 1978 -.0174 (1.81) -.0174 (1.89)

April 1979 -.0442 (4.60) -.0442 (4.81)

Apr-May 1980 -.0338 (3.51) -.0337 (3.67)

Mar-Apr 1981 -.0313 (3.37) -.0460 (5.01)

Mar-May 1982 .0304 (3.43) .0048 (0.56) .1585 (5.29)

October 1982 -.0355 (3.79) -.0571 (6.17) .0529 (1.69)

Mar-Apr 1983 -.0458 (4.91) -.0720 (7.81) .0855 (2.74)

Sep-Nov 1983 -.0740 (7.90) -.1117 (12.06) .1524 (4.87)

Mar-Apr 1984 -.0375 (4.02) -.0692 (7.49) .1385 (4.44)

Oct-Nov 1984 -.0319 (3.44) -.0580 (6.32) .1005 (3.22)

Mar-Apr 1985 .0002 (0.03) -.0340 (3.71) .2074 (6.64)

Oct-Nov 1985 -.1051 (11.86) -.1278 (13.88) -.1472 (5.75)

Mar-Apr 1986 .0098 (1.11) -.0318 (3.46) .0228 (0.89)

Oct-Nov 1986 -.0603 (6.80) -.1173 (12.73) -.0020 (0.08)

April 1987 .0197 (2.22) -.0254 (2.74) .0407 (1.59)

Oct-Nov 1987 -.0723 (8.12) -.1274 (13.71) -.0256 (1.00)

Oct-Nov 1987 .0554 (5.87) .0012 (0.13) .1035 (3.93)

Mar-Apr 1988 .0287 (3.24) -.0034 (0.37) .0127 (0.50)

Mar-Apr 1989 .0513 (5.78) .0117 (1.27) .0567 (2.22)

July 1989 .0406 (4.58) .0051 (0.56) .0354 (1.38)

Oct-Nov 1989 .0653 (8.59) .0318 (4.13) .0528 (2.21)

Mar-Apr 1990 .0880 (9.93) .0665 (7.23) .0416 (1.63)

Oct-Nov 1990 -.0023 (0.27) -.0383 (4.31) .0064 (0.25)

Dec 1990 .1579 (15.71) .1069 (10.18) .2153 (7.80)

April 1991 .0408 (4.74) .0202 (2.29) .0010 (0.04)

Oct-Nov 1991 .0290 (3.42) .0032 (0.36) -.0171 (0.69)

Mar-Apr 1992 .0134 (1.59) -.0191 (2.16) -.0147 (0.59)

Apr-May 1992 .0078 (0.91) -.0057 (0.65) -.0558 (2.18)

Apr-May 1992 -.0613 (5.35) -.0158 (1.30) -.3274 (11.02)

Sep-Oct 1992 .0166 (1.96) -.0130 (1.47) -.0200 (0.81)

November 1992 -.0194 (2.25) -.0288 (3.26) -.0973 (3.81)

Mar-Apr 1993 -.0219 (2.59) -.0307 (3.47) -.1106 (4.46)

Oct-Nov 1993 -.0290 (3.43) -.0430 (4.87) -.1045 (4.22)

Mar-May 1994 -.0021 (0.25) -.0108 (1.24) -.0899 (3.63)

Nov-Dec 1994 -.0072 (0.85) -.0233 (2.63) -.0780 (3.15)

Apr-May 1995 -.0077 (0.96) -.0131 (1.48) -.1214 (5.12)

Feb-Apr 1996 .0112 (1.44) -.0584 (7.06) .0031 (0.13)

Mar-Apr 1997 -.0346 (4.22) -.0737 (8.33) -.0920 (3.85)

Apr-May 1998 -.0554 (6.75) -.0683 (7.72) -.1563 (6.54)

Oct-Nov 1999 -.0036 (0.44) -.0329 (3.72) -.0772 (3.23)

Nov-Dec 1999 .1295 (15.78) .0941 (10.64) .0660 (2.76)

Apr-May 2000 -.0685 (8.34) -.1127 (12.73) -.1167 (4.88)

Nov-Dec 2000 .0081 (1.00) -.0074 (0.84) -.0907 (3.83)

Jan-Feb 2001 .0245 (3.02) .0202 (2.28) -.0907 (3.83)

Apr-May 2001 .0063 (0.77) -.0072 (0.82) -.0933 (3.90)

Sep-Oct 2001 .1219 (14.83) .0780 (8.80) .0723 (3.02)

Oct-Nov 2001 .0127 (1.55) -.0035 (0.40) -.0826 (3.45)

Mar-May 2002 -.0012 (0.15) -.0225 (2.55) -.0878 (3.67)

Apr-Jun 2002 -.0736 (8.93) -.0869 (9.80) -.1736 (7.24)

Oct-Nov 2002 -.0443 (5.39) -.0700 (7.90) -.1235 (5.16)

Oct-Nov 2003 -.0416 (5.07) -.0733 (8.27) -.1113 (4.65)

Oct-Nov 2004 -.0001 (0.03) .0385 (4.27) -.0320 (1.35)

Nov-Dec 2004 .1315 (17.69) .2177 (24.15) .0476 (2.01) .1060 (10.93)

May-Jun 2005 -.0161 (2.20) .0354 (3.94) -.0790 (3.34) -.0092 (1.01)

Oct-Nov 2005 -.0004 (0.07) .0261 (2.91) -.0810 (3.41) .0380 (4.19)

Mar-May 2006 -.0128 (1.75) .0224 (2.49) -.0914 (3.86) .0145 (1.59)

Sep-Oct 2006 .0272 (3.71) .0393 (4.36) -.0453 (1.91) .0688 (7.50)

**Apr-May 2007 .0292 (4.01) .0404 (4.48)** -.0678 **(**2.86**)** .0869 **(**9.65**)**

**Sep-Nov 2007 .0056 (0.77) .0263 (2.92)** -.0878 **(**3.71**)** .0554 **(**6.13**)**

**Mar-May 2008 -.0144 (1.98) -.0056 (0.63)** -.0848 **(**3.58**)** .0306 **(**3.40**)**

**Oct-Nov 2008 -.0372 (5.10) -.0168 (1.87)** -.1456 **(**6.15**)** .0194 **(**2.15**)**

**Jan-Feb 2009 -.0575 (7.88) -.0065 (0.73)** -.1669 **(**7.05**)** -.0218 **(**2.42**)**

May-Jun 2009 .0073 (1.01) .0791 (8.82) -.1103 (4.66) .0309 (3.38)

Jun-Jul 2009 -.0325 (4.45) .0500 (5.55) -.1679 (7.09) -.0041 (0.45)

Oct-Nov 2009 -.0433 (5.94) .0358 (3.97) -.1561 (6.60) -.0248 (2.75)

May 2010 -.0406 (5.58) .0391 (4.34) -.1866 (7.91) -.0067 (0.75)

June 2010 -.0006 (0.09) .0624 (6.93) -.1116 (4.71) .0111 (1.14)

Nov-Dec 2010 -.0371 (5.09) .0290 (3.23) -.1881 (7.97) .0092 (1.02)

May 2011 -.0332 (4.58) .0720 (7.99) -.1797 (7.62) -.0112 (1.27)

June 2011 -.0610 (8.21) -.0739 (8.25) -.0911 (3.85) -.0348 (3.57)

November 2011 -.0613 (8.44) .0100 (1.11) -.2389 (10.12) -.0014 (0.16)

May 2012 -.0559 (7.67) .0359 (3.99) -.2600 (11.02) .0020 (0.23)

June 2012 .2853 (38.27) .1771 (19.63) .1908 (8.05) .4466 (45.63)

November 2012 -.0927 (12.84) .0015 (0.18) -.2971 (12.60) -.0319 (3.65)

May 2013 -.0774 (10.71) .0100 (1.12) -.2735 (11.58) -.0160 (1.82)

May-Jun 2013 -.0426 (5.77) .0363 (4.03) -.2259 (9.54) .0081 (0.85)

November 2013 -.0855 (11.82) .0047 (0.52) -.2520 (10.69) -.0460 (5.21)

Nov-Dec 2013 .0401 (5.43) .0890 (9.93) -.1127 (4.76) .0966 (10.16)

January 2014 -.0242 (3.27) .0441 (4.91) -.1941 (8.20) .0268 (2.82)

March 2014 -.0456 (6.18) .0342 (3.81) -.2297 (9.71) .0048 (0.51)

May-Jun 2014 .0390 (5.41) .0856 (9.54) -.1219 (5.17) .1075 (12.26)

June 2014 -.0441 (5.97) -.0082 (0.92) -.2084 (8.81) .0315 (3.33)

September 2014 .0692 (9.38) .0791 (8.80) -.0830 (3.51) .1589 (16.81)

October 2014 .0772 (10.45) .0783 (8.70) -.0802 (3.39) .1775 (18.73)

November 2014 .0158 (2.21) .0713 (7.93) -.1847 (7.83) .0996 (11.51)

Nov-Dec 2014 .0543 (7.34) .0720 (8.01) -.1021 (4.31) .1400 (14.73)

Feb-Mar 2015 .0520 (7.05) .1002 (11.17) -.1213 (5.12) .1224 (12.94)

March 2015 .0912 (12.36) .0954 (10.60) -.0376 (1.59) .1710 (18.07)

May 2015 .0286 (3.97) .0796 (8.85) -.1452 (6.14) .1025 (11.81)

May-Jun 2015 .1179 (15.93) .1358 (15.06) -.0340 (1.44) .2007 (21.10)

October 2015 .1135 (15.36) .1208 (13.46) -.0484 (2.05) .2119 (22.25)

November 2015 .0501 (6.94) .1124 (12.50) -.1546 (6.53) .1306 (15.04)

Nov-Dec 2015 .1118 (15.10) .1485 (16.48) -.0424 (1.79) .1800 (18.91)

April 2016 .1091 (15.45) .0920 (11.15) -.0550 (2.36) .2289 (26.07)

May 2016 .0485 (6.73) .1049 (11.68) -.1345 (5.68) .1231 (14.19)

June 2016 .1525 (20.62) .1518 (16.86) -.0167 (0.71) .2624 (27.56)

Sep-Oct 2016 .0728 (9.84) .0899 (9.98) -.0873 (3.69) .1617 (16.99)

November 2016 .0493 (6.84) .1075 (11.94) -.1369 (5.79) .1247 (14.35)

Nov-Dec 2016 .1023 (13.84) .1189 (13.25) -.0479 (2.03) .1851 (19.46)

March 2017 .1126 (15.23) .1194 (13.27) -.0392 (1.66) .2047 (21.58)

April 2017 .1076 (14.61) .1021 (11.43) -.0457 (1.93) .2109 (22.35)

May 2017 .0851 (11.81) .1146 (12.80) -.0760 (3.21) .1660 (19.14)

June 2017 .1075 (14.55) .1153 (12.84) -.0311 (1.32) .1904 (20.03)

Sep-Oct 2017 .1469 (19.87) .1458 (16.20) .0204 (0.86) .2297 (24.19)

October 2017 .1233 (16.69) .1280 (14.25) -.0138 (0.59) .2079 (21.89)

November 2017 .0882 (12.24) .1007 (11.23) -.0591 (2.50) .1730 (19.97)

December 2017 .1180 (15.96) .0914 (10.17) -.0104 (0.44) .2238 (23.54)

March 2018 .0965 (13.40) .1027 (11.44) -.0588 (2.49) .1888 (21.75)

April 2018 .0984 (13.28) .0861 (9.50) -.0455 (1.92) .2015 (21.18)

June-July 2018 .1258 (17.00) .1101 (12.13) -.0087 (0.37) .2258 (23.78)

September 2018 .0878 (11.84) .0784 (8.63) -.0645 (2.73) .1936 (20.36)

Oct-Nov 2018 .0565 (7.62) .0473 (5.20) -.0944 (3.99) .1614 (16.94)

November 2018 .0901 (12.48) .0800 (8.82) -.0556 (2.35) .1872 (21.55)

December 2018 .1064 (14.37) .0870 (9.60) -.0364 (1.54) .2146 (22.58)

March 2019 .1157 (15.62) .1150 (12.69) -.0494 (2.09) .2225 (23.42)

April 2019 .1279 (17.29) .1260 (13.93) -.0242 (1.02) .2275 (23.96)

May 2019 .1297 (17.50) .1245 (13.71) -.0174 (0.74) .2287 (24.06)

June-July 2019 .1132 (15.66) .1066 (11.74) -.0265 (1.12) .2056 (23.65)

September 2019 .1416 (19.12) .1216 (13.44) .0039 (0.17) .2470 (25.97)

October 2019 .1324 (17.88) .1284 (14.17) -.0210 (0.89) .2343 (24.70)

Nov-Dec 2019 .1073 (14.86) .0917 (10.10) -.0397 (1.68) .2081 (23.95)

December 2019 .1423 (19.21) .1170 (12.89) .0079 (0.34) .2497 (26.30)

**July-August 2020 .1204 (16.71) .0927 (10.25) -.0347 (1.47) .2313 (26.76)**

**Aug-Sep 2020 .1165 (15.80) .0418 (4.66) -.0587 (2.49) .2907 (30.82)**

**Oct-Nov 2020 .0389 (5.25) -.0093 (1.00) -.1206 (5.10) .1764 (18.70)**

**Feb-March 2021 .0161 (2.29) -.0478 (5.35) -.1819 (7.82) .1726 (20.42)**

**Mar-Apr 2021 .0405 (5.44) -.0149 (1.59) -.1214 (5.13) .1846 (19.50)**

April-May 2021 .1030 (14.49) .0496 (5.48) -.0803 (3.45) .2472 (28.94)

June-July 2021 .1204 (16.94) .0720 (7.92) -.0677 (2.90) .2618 (30.82)

Sep-Oct 2021 .1940 (26.03) .1101 (11.93) -.0124 (0.52) .3678 (39.64)

Jan-Feb 2022 .0818 (11.53) .0419 (4.64) -.0891 (3.83) .2111 (24.83)

April-May 2022 .1328 (17.82) .0765 (8.14) -.0082 (0.35) .2642 (27.89)

June-July 2022 .0924 (13.00) .0738 (8.13) -.0625 (2.68) .2018 (23.74)

Jan-Feb 2023 .0983 (13.81) .0545 (6.00) -.0630 (2.70) .2242 (26.38)

Country dummies 40 8 9 20

Cons 2.8931 2.8919 2.5529 2.8527

Adjusted R^2^ .1699 .1263 .1883 .0830

N 3,226,021 1,336,185 796,865 1,092,971

All equations include a female dummy. Excluded category for columns 1 & 2 is Sept-Oct 1973. For column 3 it is March-April 1981. Countries distribution by year below with sample sizes in parentheses based on life satisfaction not missing. Numbers below relate to observations where life satisfaction present. T-statistics in parentheses

**1. Nine Western European countries (1973-2023) – n=1,336,452**

Belgium (145,750); France (145,414); Denmark (143,604); Germany (221,516); Ireland (143,367); Italy (148,373); Luxembourg (66972); Netherlands (144,298) and UK (177,158).

**2. Southern & Northern (1982-2023) – n=796,984**

Greece 1981-2023 (130,724); Spain (124,203); Portugal (125,341); Finland (100,729); Sweden (100,822); Austria (99,842); Cyprus (42,991); Malta (42479) Norway (16,297); Switzerland (6,338); Iceland (7,580).

**3. Eastern (2004-2023) – n=1,089,857**

Czechia (86,543); Estonia (83,271); Hungary (85,796); Latvia (84,006); Lithuania (83,977); Malta (42,479); Poland (82,289); Slovakia (85,803); Slovenia (85,160); Bulgaria (83,863); Romania (85,863); Turkey (40,126); Croatia (79,429); Turkish Cyprus (20,913); N Macedonia (36082); Montenegro (14,489); Serbia (21,241); Albania (17,534); Bosnia/Herzegovina (6,074); Kosovo (6,344); Moldova (1,0 06)
