# Supplementary material for: Were COVID and the Great Recession well-being reducing?
Source: PLoS One. 2024 Nov 27;19(11):e0305347. doi: 10.1371/journal.pone.0305347 (PMC11602031; doi:10.1371/journal.pone.0305347)
Supplement: S5 Table — (DOCX) [file pone.0305347.s005.docx]

Appendix Table S5. EU changes in expectations and the unemployment rate 2007-2009

Financial General economic Unemployment Unemployment

situation situation expectations rate

Jun-07 1.1 -2.7 5.8 7.5

Jul-07 1.1 -3.3 3.5 7.5

Aug-07 -0.3 -6.8 6.2 7.5

Sep-07 -1.7 -10.2 8.5 7.4

Oct-07 -1.6 -9.1 7.2 7.4

Nov-07 -2.9 -13.9 8.4 7.3

Dec-07 -3.4 -12.8 7.6 7.3

Jan-08 -4.8 -17.5 10.1 7.2

Feb-08 -4.3 -17.6 12.1 7.1

Mar-08 -4.5 -17.6 13.3 7.1

Apr-08 -5.1 -19.3 13.1 7.1

May-08 -6.4 -21.2 15.9 7.2

Jun-08 -10.3 -26.6 17.8 7.3

Jul-08 -11.0 -31.7 23.5 7.2

Aug-08 -8.7 -28.4 25.8 7.3

Sep-08 -7.5 -26.2 27.0 7.3

Oct-08 -9.3 -32.6 37.7 7.5

Nov-08 -8.5 -32.2 47.5 7.7

Dec-08 -8.8 -36.6 56.8 7.9

Jan-09 -8.1 -36.1 59.2 8.4

Feb-09 -10.2 -38.1 63.6 8.7

Mar-09 -9.2 -41.1 67.4 8.9

Apr-09 -7.5 -34.4 64.4 9.1

May-09 -6.2 -28.1 61.0 9.2
