# Supplementary material for: Were COVID and the Great Recession well-being reducing?
Source: PLoS One. 2024 Nov 27;19(11):e0305347. doi: 10.1371/journal.pone.0305347 (PMC11602031; doi:10.1371/journal.pone.0305347)
Supplement: S6 Table — (DOCX) [file pone.0305347.s006.docx]

Appendix Table S6. Unemployment expectations in 28 countries 2007-2009 and 2017-2023

**a) Western Europe**

Austria Belgium Cyprus Denmark Finland France

2007 -3 10 -27 -8 -2 5

2008 17 22 12 20 18 27

2009 52 65 73 31 43 61

2019 8 10 -7 1 6 9

2020 27 51 47 15 29 45

2021 4 20 34 -6 4 27

2022 17 18 27 14 11 9

2023 13 19 16 23 12 15

Germany Greece Ireland Italy Luxembourg Malta

2007 -1 35 33 19 5 -14

2008 17 50 54 27 5 -6

2009 70 63 63 43 31 38

2019 16 7 7 14 10 -23

2020 44 52 26 41 49 14

2021 25 45 -3 29 26 -11

2022 28 36 12 29 17 -1

2023 20 18 17 14 22 5

Netherlands Portugal Spain Sweden UK

2007 -16 43 12 -18 28

2008 11 51 46 29 45

2009 61 64 42 39 55

2017 -23 5 -3 16 16

2018 -26 -11 -1 3 19

2019 -7 -1 13 2 24

2020 52 53 48 19 43

2021 10 30 18 -2 n/a

2022 -3 26 19 18 n/a

2023 0 32 18 41 n/a

**b) Eastern Europe**

Romania Bulgaria Croatia Czechia Estonia Hungary

2007 16 10 41 3 -7 53

2008 21 17 37 14 34 53

2009 69 55 56 45 47 71

2019 16 15 -1 10 6 -2

2020 28 38 32 42 34 32

2021 n/a 30 24 26 20 23

2022 n/a 29 16 29 36 40

2023 n/a 23 3 31 48 41

Latvia Lithuania Poland Slovenia Slovakia Turkey

2007 -4 21 31 11 -12 19

2008 32 29 16 19 1 37

2009 66 62 21 54 53 33

2019 6 3 -2 7 2 42

2020 29 29 39 45 47 40

2021 24 20 28 29 36 33

2022 26 26 31 24 26 31

2023 23 19 28 22 26 23

Albania Serbia N Macedonia Montenegro

2019 4 -25 -5 10

2020 19 -14 17 25

2021 12 -10 20 18

2022 11 -7 18 20

2023 6 -7 0 6

Source: EU Commission
